# Epigallocatechin gallate improves neuronal damage in animal model of ischemic stroke and glutamate-exposed neurons via modulation of hippocalcin expression

**DOI:** 10.1371/journal.pone.0299042

**Published:** 2024-03-01

**Authors:** Dong-Ju Park, Ju-Bin Kang, Phil-Ok Koh

**Affiliations:** Department of Anatomy, College of Veterinary Medicine, Research Institute of Life Science, Gyeongsang National University, Jinju, South Korea; Helwan University, EGYPT

## Abstract

Epigallocatechin gallate (EGCG) is a polyphenolic component of green tea that has anti-oxidative and anti-inflammatory effects in neurons. Ischemic stroke is a major neurological disease that causes irreversible brain disorders. It increases the intracellular calcium concentration and induces apoptosis. The regulation of intracellular calcium concentration is important to maintain the function of the nervous system. Hippocalcin is a neuronal calcium sensor protein that controls intracellular calcium concentration. We investigated whether EGCG treatment regulates the expression of hippocalcin in stroke animal model and glutamate-induced neuronal damage. We performed middle cerebral artery occlusion (MCAO) to induce cerebral ischemia. EGCG (50 mg/kg) or phosphate buffered saline was injected into the abdominal cavity just before MCAO surgery. The neurobehavioral tests were performed 24 h after MCAO surgery and cerebral cortex tissue was collected. MCAO damage induced severe neurobehavioral disorders, increased infarct volume, and decreased the expression of hippocalcin in the cerebral cortex. However, EGCG treatment improved these deficits and alleviated the decrease in hippocalcin expression in cerebral cortex. In addition, EGCG dose-dependently alleviated neuronal cell death and intracellular calcium overload in glutamate-exposed neurons. Glutamate exposure reduced hippocalcin expression, decreased Bcl-2 expression, and increased Bax expression. However, EGCG treatment mitigated these changes caused by glutamate toxicity. EGCG also attenuated the increase in caspase-3 and cleaved caspase-3 expressions caused by glutamate exposure. The effect of EGCG was more pronounced in non-transfected cells than in hippocalcin siRNA-transfected cells. These findings demonstrate that EGCG protects neurons against glutamate toxicity through the regulation of Bcl-2 family proteins and caspase-3. It is known that hippocalcin exerts anti-apoptotic effect through the modulation of apoptotic pathway. Thus, we can suggest evidence that EGCG has a neuroprotective effect by regulating hippocalcin expression in ischemic brain damage and glutamate-exposed cells.

## Introduction

Stroke is one of the leading causes of death and disability. Stroke can be divided into two subtypes: ischemic stroke and hemorrhagic stroke [1]. Ischemic stroke is the most common form of stroke caused by blockage of blood vessels in the brain [2]. It leads to a lack of oxygen and glucose, a depletion of energy, an excessive release of the excitatory neurotransmitter glutamate, and an intracellular calcium overload in the brain, leading to nerve damage and death [3].

In nervous system, calcium mediates specific processes including neurotransmission, synaptic modification, and neurofilament stabilization [4–7]. Imbalance of intracellular calcium ions due to oxidative stress induces activation of calcium-dependent enzymes and causes neuronal cell death [8, 9]. Thus, the maintenance of intracellular calcium level is very important for cell survival. Calcium binding proteins play a critical role for the maintenance of intracellular calcium levels. They have high calcium binding capacity and regulates calcium levels [10]. Hippocalcin is one of the calcium-binding proteins that regulate calcium levels. Hippocalcin has a high affinity for calcium ions and contains three calcium-binding EF-hand motifs [11]. It exists in the central nervous system and regulates the electrical signaling of neurons by controlling the intracellular calcium concentration [12]. It is abundant in pyramidal cells of the hippocampus and is also found in the hypothalamic region [11]. Hippocalcin has several functions in synaptic plasticity, cellular differentiation, and anti-apoptosis [13, 14]. Hippocalcin also regulates neuronal differentiation and neuronal excitability [15, 16]. Hippocalcin preserves neurons from apoptotic damage by inhibiting calcium overload, which prevents caspase-3-dependent and caspase-3-independent apoptotic pathways [14]. It also inhibits caspase-12 activity, reduces endoplasmic reticulum stress, and attenuates neuronal cell death [17]. In particular, hippocalcin performs anti-apoptotic functions by interacting with neuronal apoptosis inhibitory protein (NAIP) [14]. It also exerts neuroprotective effects in neurological disorders such as hypothalamic dysfunction and Alzheimer’s disease [18, 19]. Hippocalcin has neuroprotective effects by alleviating amyloid beta toxicity in Alzheimer’s disease and by improving dysregulation of intracellular calcium levels [18]. Moreover, hippocalcin plays a neuroprotective role by its anti-apoptotic function in hypothalamic damage caused by heat stroke [19].

Epigallocatechin gallate (EGCG) is a major polyphenol that exists in green tea [20]. EGCG protects cells against harmful factors such as oxidative stress, carcinogens, inflammatory factors, and neurotoxicity [21–23]. It acts as a powerful antioxidant in the nervous system, regulates apoptosis-related signaling pathways, and promotes neuronal regeneration [24]. Furthermore, EGCG prevents generation of reactive oxygen species and increase in intracellular calcium [25]. Neuronal stem cells play a pivotal role in neuronal regeneration and plasticity, and contribute to recovery from nerve damage and neurodegeneration. In particular, previous studies have shown that hippocalcin plays an important role in determining the cell fate of neural stem cells, promoting neuronal differentiation and neurogenesis [16] Although the neuroprotective effects of EGCG in neuronal damage have been reported, the regulation of intracellular calcium concentration by EGCG in ischemic brain damage has not been clearly elucidated. We hypothesized that EGCG administration may affect changes in hippocalcin in ischemic stroke. Thus, we investigated whether EGCG has a neuroprotective effect through the regulation of intracellular calcium concentration and hippocalcin expression in animal model of ischemic stroke and glutamate-exposed cells.

## Materials and methods

### Experimental animals preparation and drug administration

Sprague-Dawley rats (Male, n = 60, 210–220 g) were supplied from Samtako Co. (Animal Breeding Center, Osan, Korea). Animals were acclimatized for seven days and kept under conditions with a temperature range of 22°C ± 1°C and 12 h light/12 h dark lighting cycle. Animals were randomly selected and grouped into four groups as follows: phosphate buffered saline (PBS) + Sham, EGCG + Sham, PBS + MCAO, and EGCG + MCAO (n = 15 per group). EGCG (Sigma Aidrich, St. Louis, MO, USA) was completely dissolved in PBS by vortexing. EGCG (50 mg/kg) or PBS was treated by intraperitoneal injection just before the surgical procedure [26, 27]. All experimental procedures were approved and carried out in accordance with the guidelines of the Institutional Animal Care and Use Committee of Gyeongsang National University (No. GNU-1902180-R0008).

### Middle cerebral artery occlusion

Animals were anesthetized by intraperitoneal injection with Zoletil (50 mg/kg, Virbac, Carros, France) and kept in a ventral position on a surgical table. MCAO surgical process was performed as a previously described method [28]. Briefly, a midline incision was made on ventral cervical skin and muscle tissue was dissected to expose the right carotid artery sheath. Common carotid artery (CCA) was separated from vagus nerve and surrounding tissue, external carotid artery (ECA) and internal carotid artery (ICA) were exposed. A 4/0 monofilament nylon with a flame-bulged tip was inserted to the stump of ECA and proceeded to ICA. Nylon was inserted until resistance was felt. The length of inserted nylon was approximately 22 to 24 mm. Inserted nylon was fixed with ECA and skin was sutured with 3/0 black silk. To prevent hypothermia, animals were placed on a heating pad until they completely recovered from anesthesia. The neurological deficit scoring test and corner test were performed 24 h after MCAO surgery. After anesthesia with Zoletil (Virbac), animals were quickly decapitated and sacrificed for further experimentation. We tried to minimize pain to the animals.

### Neurological deficit scoring test

Animals were subjected to a neurological deficit scoring test to evaluate neurological impairments. They were individually housed in cage and behavioral scores were recorded. Animals were observed and graded by five-point scale based on the following criteria: no deficit (0), weakness and incomplete extension of contralateral forelimb (1), repetitive circling behavior in contralateral direction (2), falling down, hypertensive reflex, and seizure (3), loss of voluntary movement and unconsciousness (4).

### Corner test

Corner test was performed to evaluate postural asymmetries and sensorimotor dysfunction according to the previously described method [29]. Two boards (30 x 20 x 1 cm^3^) were placed at an angle of 30° with a small opening, inducing the animals to enter the corner. When the animals reached to the corner, both vibrissae and skin were stimulated by two boards and animals turned back from the corner. The number of right and left turns were recorded and only a complete turn was considered. The right side represents the ipsilateral side of the MCAO region. Animals were trained for seven days before MCAO surgery and animals with similar rates in the left and right turns were used for this experiment. The results of the experiment are displayed as the number of right or left turns.

### Triphenyltetrazolium chloride staining

Triphenyltetrazolium chloride (TTC) staining was conducted to detect the infarcted area from brain tissue [30]. Whole brain was carefully removed from skull and placed in the brain slicer matrix (Ted Pella, Redding, CA, USA). Brain was sliced at 2 mm intervals in coronal section. Sections were stained in 1% TTC (Sigma Aldrich) for 20 min at 37°C. They were fixed in 4% neutral buffered paraformaldehyde (NBP) for 24 h and all serial sections were scanned by Agfar ARCUS 1200TM (Agfar Gevaert, Mortsel, Belgium). Intact areas were stained with TTC and appeared red color, while infarct areas were un-stained and appeared white color. Serial section images were analyzed by using Image-ProPlus 4.0 software (Media Cybernetics, Bethesda, MD, USA). The volume of infarction was calculated as the infarction area/whole section area ×100%.

### Two-dimensional gel electrophoresis

The right cerebral cortex was homogenated with lysis buffer [8 M urea, 4% CHAPS, ampholytes, and 40 mM Tris-HCl (pH 8.0)] and mixed with 20% trichloroacetic acid. The mixtures were centrifuged at 16,000 g for 10 min at 4°C. Supernatants were removed and pellets were resuspended with lysis buffer. Protein concentration was measured by Bradford protein assay kit (Bio-Rad, Hercules CA, USA) following the manufacturer’s instruction. First dimensional separation was conducted by isoelectric focusing (IEF) method. Immobilized pH gradient (IPG) gel strips (17 cm, pH 4–7, Bio-Rad) were rehydrated with rehydration buffer (8 M urea, 2% CHAPS, 20 mM DTT, 0.5% IPG buffer, and bromophenol blue) containing total protein sample (50 μg) for 15 h at room temperature. Rehydrated IPG strips were kept in Ettan IPGphor 3 System (GE Healthcare, Uppsala, Sweden) with the following IEF conditions: 250 V for 15 min, 10,000 V for 3 h, and then 10,000 V to 50,000 V. IPG strips were reacted with equilibration buffer [6 M urea, 30% glycerol, 2% sodium dodecyl sulfate, and 50 mM Tris-HCl (pH 8.8)] containing reductant (1% DTT) for 10 min and subsequently reacted with same equilibration buffer containing alkylator (2.5% iodoacetamide) for 10 min. They were placed on 7.5–17.5% gradient gels, covered with 0.5% agarose gel, and electrophoresed using Protein-II XI electrophoresis equipment (Bio-Rad). Electrophoresis was performed by the following condition: 5 mA for 2 h and 10 mA for 10 h at 10°C. It was performed until the bromophenol blue dye moved to the bottom of the gel. Gels were carefully removed from electrophoresis equipment, immersed with a fixation solution (12% acetic acid and 50% methanol) for 2 h, stained with a silver staining solution (0.2% silver nitrate) for 20 min, and immersed in a 2% sodium carbonate solution for protein spot image development. Stained gels were scanned with Agfa ARCUS 1200^TM^ (Agfa-Gevaert, Mortsel, Belgium) and analyzed by PDQuest 2-D analysis software (Bio-Rad). Protein spots with a difference in intensities of experimental group were detected. These spots were separated from the gel. Isolated gel pieces were reacted with a destaining solution (30 mM potassium hexacyanoferrate, 100 mM sodium thiosulfate) and digested with trypsin buffer. Extracted protein samples were analyzed using Voyager System DE-STR MALDI-TOF mass spectrometer (Applied Biosystem, Foster City, CA, USA) and obtained peptide peaks were identified based on MS-Fit and ProFound programs, SWISS-Prot and National Center for Biotechnology Information database. Hippocalcin protein level was determined as the ratio of spot intensity in each group to spot intensity in the PBS + Sham animals. Hippocalcin levels in PBS + Sham animals were represented to 1.

### Reverse transcription-polymerase chain reaction

Right cerebral cortical tissues were lysed and homogenized with Trizol Reagent (Life Technologies, Rockville, MD, USA). Homogenates were centrifuged at 12,000 g for 5 min at 4°C. Supernatants were collected and total RNA was isolated with isopropanol. Total RNA (1 ug) was converted into a single-stranded complementary DNA (cDNA) using a Superscript III first-strand system (Invitrogen, Carlsbad, CA, USA). The target gene was detected from the cDNA template by a specific primer sequence as follows: hippocalcin, forward primer: 5’-ACGCCAACTTCTTCCCCTATG-3’, reverse primer: 5’-AGCCATCAGCGTCTTTGTTT-3’, β-actin, forward primer: 5’-GGGTCAGAAGGACTCCTACG-3’, reverse primer: 5’-GGTCTCAAACATGATCTGGG-3’. PCR amplification was performed in 30 cycles and carried out as follows: denaturation of the template for 30 sec at 95°C, annealing of the primers for 30 sec at 52.5°C, and extension for 30 sec at 72°C. Denaturation at 95°C for 5 min and final extension at 72°C for 5 min were performed before and after the amplification process, respectively. Amplified DNA was mixed with a Loading STAR dye (Dyne Bio, Seongnam, Korea), loaded into a 1% agarose gel, and electrophoresed using Mupid-2plus (Takara bio, Shiga, Japan) for 15 min until loading dye reached the bottom of gel. PCR products were visualized under UV light and captured. Intensities of PCR products were calculated with SigmaGel 1.0 densitometry software (Jandel Scientific) and SigmaPlot 4.0 (SPSS Inc.). Densitometric analyses from reverse transcription-PCR are represented as a ratio of hippocalcin intensity to β-actin intensity.

### Western blot analysis

Right cerebral cortical tissues and cultured cell samples were homogenized with lysis buffer [1% Triton X-100, 1 mM EDTA in PBS (pH 7.4)] containing 200 mM phenylmethylsulfonyl fluoride. Homogenates were dispersed by sonication and centrifuged at 15,000 g for 20 min at 4°C. Supernatants were collected and frozen at -70°C for further experiment. Protein concentration was measured with a bicinchoninic acid protein assay kit (Thermo Fisher Scientific, Waltham, MA, USA) by following the manufacturer’s instructions. Protein samples (10 μg) were denatured by heating for 5 min at 100°C, loaded into 10% sodium dodecyl sulfate-polyacrylamide gels, and electrophoresed. Separated protein samples were transferred from gels to polyvinylidene fluoride membranes (Millipore, Billerica, MA, USA). Membranes were incubated with 5% skim milk in Tris-buffered saline containing 0.1% Tween-20 (TBST) for 1 h to block non-specific bindings. They were washed with TBST three times for 10 min and incubated with primary antibodies on a shaker for overnight at 4°C. The following antibodies were used: anti-hippocalcin (1:1,000 dilution, rabbit IgG, Thermo Fisher Scientific), anti-Bcl-2 (1:1,000 dilution, mouse IgG, Santa Cruz Biotechnology, Santa Cruz, CA, USA), anti-Bax (1:1,000 dilution, mouse IgG, Santa Cruz Biotechnology), anti-caspase-3 (1:1,000 dilution, rabbit IgG, Cell Signaling Technology, Danvers, MA, USA), anti-cleaved caspase-3 (1:1,000 dilution, rabbit IgG, Cell Signaling Technology), and anti-β-actin (1:1,000 dilution, mouse IgG, Santa Cruz Biotechnology). Membranes were rinsed with TBST three times for 10 min and incubated with horseradish peroxidase-conjugated anti-rabbit IgG (1:5,000, Promega, Madison, WI, USA) or anti-mouse IgG (1:5,000, Thermo Fisher Scientific). They were rinsed with TBST three times for 10 min and immersed with enhanced chemiluminescence detection reagents (GE Healthcare, Little Chalfont, UK). Membranes were placed in an X-ray film cassette (Advansta Inc., San Jose, CA, USA) and visualized by exposure to Fuji medical X-ray film (Fuji Film, Tokyo, Japan). Band intensities were evaluated using SigmaGel 1.0 densitometry software (Jandel Scientific, San Rafael, CA, USA) and SigmaPlot 4.0 (SPSS Inc., Point Richmond, CA, USA). Densitometric analyses from Western blot are represented as a ratio of hippocalcin intensity to β-actin intensity. Hippocalcin levels in PBS + Sham animals were represented to 1.

### Hippocampal cell line culture

Mouse hippocampal HT22 cell was cultured in L-glutamate-free Dulbecco’s modified Eagle’s medium (DMEM, Gibco BRL, Gaithersburg, MD, USA) with heat-inactivated 10% fetal bovine serum and 1% antibiotic cocktail (100 mg/ml streptomycin and 100 units/ml penicillin). Cells were cultured in 5% CO_2_ condition at 37°C and passaged at least three times a week for 5 to 10 passages. When confluency reached 70%, cells were treated with 5 mM glutamate (Sigma Aidrich) to induce glutamate neurotoxicity [31]. EGCG (10, 20, and 40 μM) were dissolved in PBS and treated 1 h before glutamate treatment [32]. After 24 h of glutamate treatment, the medium was discarded and cells were collected and stored at -70°C for further experiment.

### Cell viability test

3-(4,5-Dimethythiazol-2-yl)-2,5-diphenyl tetrazolium bromide (MTT) colorimetric assay was performed to access cell viability. HT22 cells were seeded in 96-well microplates at a seeding density of 5 × 10^3^ cells/ml with DMEM containing serum for 24 h. EGCG and/or glutamate were treated according to previous described method. Medium was changed to serum-free DMEM and MTT solution (Sigma Aldrich) was treated with a final concentration of 5 mg/ml and incubated for 4 h at 37°C. After removing the medium, dimethyl sulfoxide was added to dissolve the MTT formazan. Samples were collected from each well and optical density (OD) was read at 570 nm. Results of cell viability were expressed by the following formula: mean OD of each group of cells/ mean OD of PBS-treated cells ×100%. The cell viability of PBS-treated group was set at 100%.

### Intracellular calcium concentration measurement

Fluo-3-acetoxymethyl (Fluo-3-AM, Thermo Fisher Scientific) was used as a calcium indicator to quantify the intracellular calcium concentration with flow cytometry in living cells [33]. HT22 cells were stained with Fluo-3 AM (3 μM) in DMEM for 1 h at 37°C, dissociated with trypsin, and washed with Locke’s solution (pH 7.3). Washed cells were centrifuged at 1,000 g for 10 min and resuspended in Locke’s solution with a final concentration of 1 × 10^6^ cells/ml. Cells were gently resuspended immediately before applying to FACSVerse Flow Cytometer (BD Biosciences, San Jose, CA, USA). Fluo-3 AM fluorescence was excited at 488 nm wavelength laser and emission wavelength was detected at 530 ± 20 nm. Results were analyzed with FACSuite software (BD Biosciences) and final calcium concentration was determined by the following formula [Ca^2+^]i = K_d_ × (F—F_min_)/ (F_max_—F)). K_d_ value of Fluo-3 AM for Ca^2+^ was determined to be 400 nM which is a physiological ionic strength in vertebrates. F indicates fluorescence intensity of all samples at 530 ± 20 nm. F_max_ represents maximal fluorescence intensity obtained by Ca^2+^ saturates with ionomycin (5 μM) in HT22 cells. F_min_ indicates minimal fluorescence intensity obtained by chelating Ca^2+^ with ethylene glycol tetraacetic acid (2 mM) in HT22 cells [34].

### Immunocytochemistry

HT22 cells were treated with EGCG and/or glutamate, fixed in a 4% NBP solution for 10 min, and rinsed with PBS. They were incubated with 5% normal goat serum for 1 h at room temperature to prevent non-specific binding to antibodies and serum was removed. Cells were incubated with rabbit-derived anti-hippocalcin antibody (1:100, Thermo Fisher Scientific) overnight at 4°C and were washed with PBS. They were incubated with fluorescein isothiocyanate (FITC)-conjugated goat-derived anti-rabbit IgG (1:200, Jackson Immuno Research Laboratories) for 2 h at room temperature and washed with PBS. Cells were stained with 4`,6-Diamidino-2-phenylindole dihydrochloride (DAPI) (Sigma Aidrich) for 10 min at room temperature and were mounted with mounting medium for fluorescence microscopy (Dako North America). Stained cells were observed using confocal laser microscopes (AXIO, Carl Zeiss Corporation). Images were analyzed with ImageJ 1.50i (Media Cybernetics). The results were expressed as the relative integrated density of each group to that of the integrated density of PBS + Sham group. Hippocalcin levels in PBS + Sham animals were represented to 1.

### Hippocalcin siRNA transfection and drug treatment

Hippocalcin siRNA (Thermo Fisher Scientific) was treated for hippocalcin gene silencing in HT22 cells. Cells were maintained in DMEM medium with serum and cell medium was changed to serum-free DMEM medium when confluency was about 70%. Lipofectamine 3000^TM^ (Thermo Fisher Scientific) was diluted with serum-free DMEM and incubated for 5 min. Diluted Lipofectamine 3000^TM^ was mixed with hippocalcin siRNA and incubated for 20 min. siRNA-lipid complex was treated to cells with a final concentration of 30 pmol and incubated for 24 h. Medium was changed and PBS or EGCG (40 μM) was treated 1 h before glutamate treatment. After 24 h of treatment with glutamate (5 mM), cells were collected.

### Statistical analysis

All numerical data obtained from this study were expressed as means ± standard error of mean (S.E.M.) for each group. Data were analyzed using Shapiro-Wilk test for normality and Levene’s test for homogeneity of variance. Statistical significance of intergroup differences was analyzed by one-way ANOVA or two-way ANOVA followed by post-hoc Scheffe’s test. Significance was analyzed by Mann-Whitney U test when data unsatisfied the normal distribution and/or homogeneity of variance. Significance of EGCG effect in a dose-dependent manner was analyzed by two-way ANOVA followed by Dunnett’s multiple comparison tests. Significance was determined by *P*-value and the significance level was set at p < 0.05.

## Results

### EGCG improves neurobehavioral disorder and cerebral infarction caused by MCAO damage

**Fig 1:**
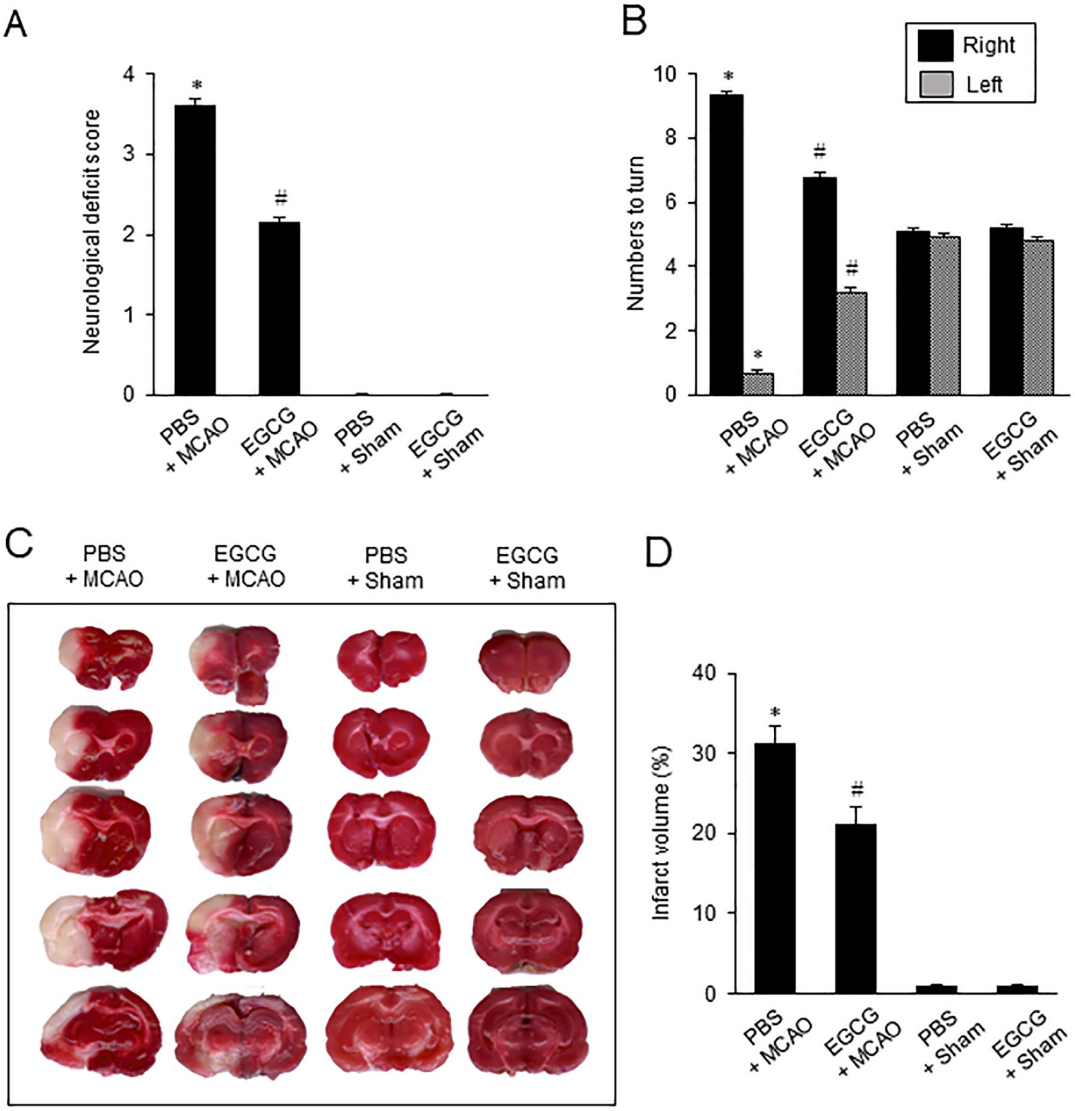
Neurobehavioral tests and infarct volume analysis. EGCG improves neurological disorders and cerebral infarctions caused by MCAO damage. Neurological deficits scoring test (A), corner test (B), TTC staining (C), and infarct volume (D) in phosphate-buffered saline (PBS) + middle cerebral artery occlusion (MCAO), epigallocatechin gallate (EGCG) + MCAO, PBS + Sham, and EGCG + Sham animals. Intact area was stained red color, while the ischemic area remained white color (C). Infarct volume was calculated by the percentage of the infarction area to the whole brain area (D). Data (A and B, *n =* 15: D, *n* = 4) are shown as means ± S.E.M. * p < 0.01, vs. PBS + Sham; # p < 0.01, vs. PBS + MCAO.

Before the investigation of hippocalcin changes by EGCG treatment in MCAO animals, we confirmed the neuroprotective effect of EGCG in MCAO damage. Neurological dysfunction was evaluated by neurological deficit scoring test and corner test. PBS + MCAO animals showed serious neurological dysfunction such as seizures or no spontaneous movement. However, EGCG treatment improved these neurological disorders. EGCG + MCAO animals showed occasional circling behavior and a forelimb flexion posture. Sham animals did not show abnormal behaviors regardless of drug treatment. Neurological function scores were 3.63 ± 0.06 in PBS + MCAO animals and 2.17 ± 0.05 in EGCG + MCAO animals (Fig 1A). Unilateral neuronal damage was analyzed by corner tests. Right-biased turning behavior was observed in PBS + MCAO animals. However, these behaviors were alleviated in EGCG + MCAO animals. The corner test was performed 10 times. The number of right turns was 9.33 ± 0.13 in PBS + MCAO animals and 6.79 ± 0.11 in EGCG + MCAO animals (Fig 1B). TTC staining results showed a large infarction in PBS + MCAO animals, but EGCG treatment reduced this infarction (Fig 1C). Infarct volume was evaluated as the ratio of infarct area to the whole brain area. It was 31.34 ± 2.12% in PBS + MCAO animals and 21.23 ± 2.08% in EGCG + MCAO animals (Fig 1D).

### EGCG alleviates the reduction in hippocalcin expression due to MCAO damage

**Fig 2:**
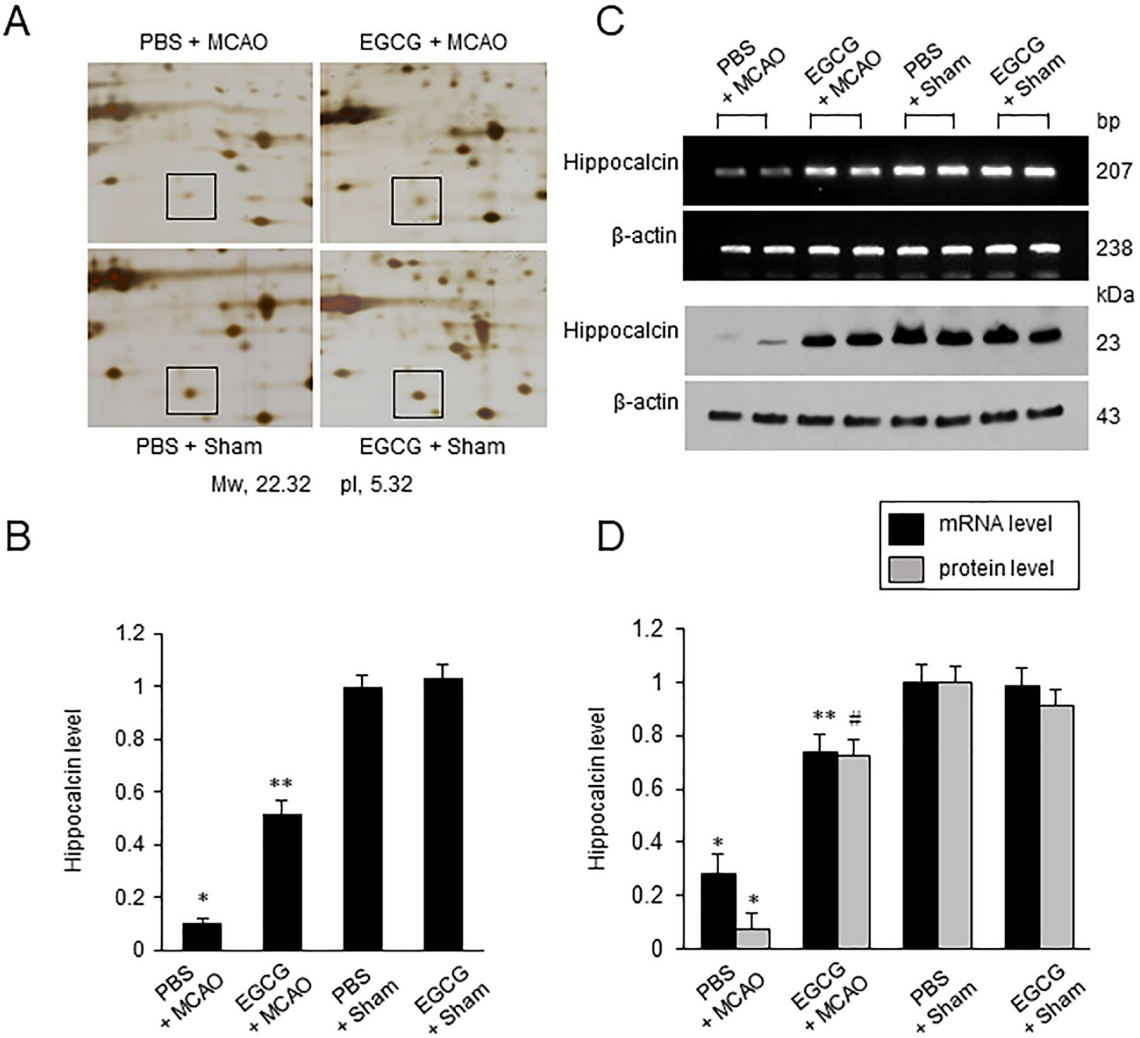
Expression change of hippocalcin in cerebral cortex of MCAO animal. EGCG alleviates the reduction in hippocalcin expression caused by MCAO damage. Proteomic study (A and B), transcription-PCR and Western blot analysis (C and D) of hippocalcin in phosphate buffered saline (PBS) + middle cerebral artery occlusion (MCAO), epigallocatechin gallate (EGCG) + MCAO, PBS + Sham, and EGCG + Sham animals. Spot intensities were measured by PDQuest software. Intensities of protein spots are represented as a ratio relative to PBS + Sham animals (B). Mw and pI indicate the molecular weight and isoelectric point, respectively. Densitometric analyses from reverse transcription-PCR and Western blot are represented as a ratio of hippocalcin intensity to β-actin intensity (D). Hippocalcin levels in PBS + Sham animals were represented to 1. Each lane represents an individual animal. Data (*n* = 4) are shown as means ± S.E.M. * p < 0.001, vs PBS + Sham; ** p < 0.01, vs. PBS + MCAO; *#* p < 0.05, vs. PBS + MCAO.

We identified the change in hippocalcin protein expression using a proteomic approach (Fig 2A). A decrease in hippocalcin expression was observed in PBS + MCAO animals, and this decrease was alleviated by EGCG treatment. The matched peptide mass was 10/102 and the sequence coverage was 52%. Hippocalcin levels in the PBS + Sham animals was set to 1. Hippocalcin levels were 0.11 ± 0.02 and 0.52 ± 0.05 in PBS + MCAO and EGCG + MCAO animals, respectively (Fig 2B). However, they were similar in sham animals regardless of PBS or EGCG treatment. Reverse transcription-PCR and Western blot analyses were performed to confirm the changes in the expression of hippocalcin (Fig 2C). EGCG administration mitigated the reduction in hippocalcin caused by MCAO damage. Hippocalcin mRNA level was 0.28 ± 0.05 in PBS + MCAO animals and 0.74 ± 0.30 in EGCG + MCAO animals (Fig 2D). Hippocalcin protein level was 0.08 ± 0.02 in PBS + MCAO animals and 0.73 ± 0.03 in EGCG + MCAO animals (Fig 2D).

### EGCG attenuates neuronal cell death and intracellular calcium concentration overload in glutamate-exposed neuron

**Fig 3:**
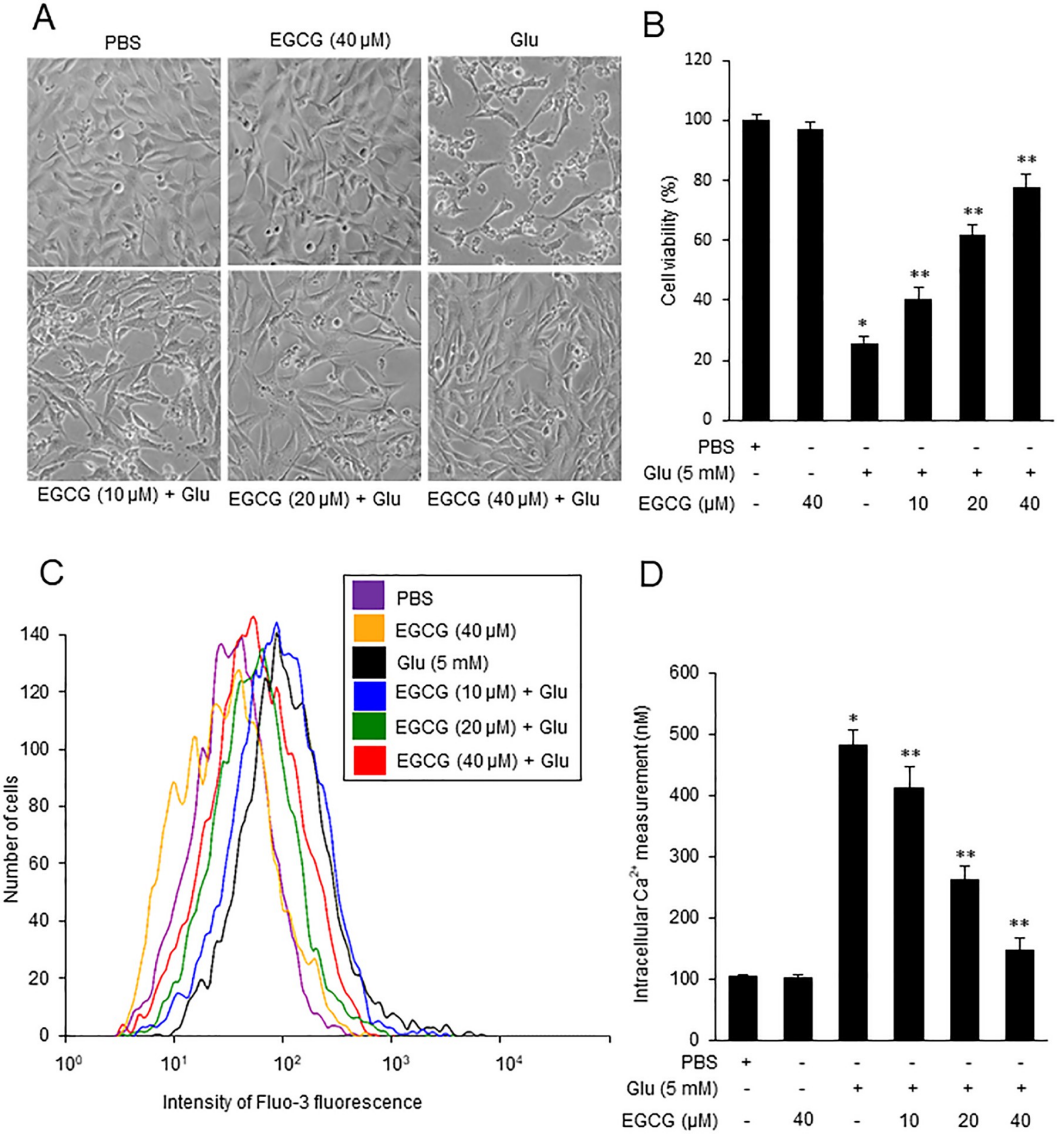
The measurement of cell viabilty and intracellular calcium concentration in nerons. EGCG alleviates neuronal cell death and intracellular calcium concentration overload in glutamate-exposed neurons. Representative photos of cells (A), cell viability (B), and intracellular calcium concentration (C and D) in glutamate (Glu)- and/or epigallocatechin gallate (EGCG)-treated hippocampal neuronal cells (HT22). HT22 cells were treated with glutamate (5 mM) for 24 h and EGCG (10, 20, and 40 μM) was treated 1 h before glutamate exposure. The cell viability of PBS-treated group was set at 100% (B). Labeled neurons with Fluo-3 AM were measured using a luminescence spectrophotometer (D). Data (*n* = 5) are presented as means ± S.E.M. * p < 0.001, vs. PBS; ** p < 0.001, vs. Glu (5 mM).

The results of MTT assay showed the protective effect of EGCG on neuronal cell damage caused by glutamate exposure. We observed severe cell death in the glutamate-treated group. EGCG treatment alleviated cell death in a dose-dependent manner (Fig 3A). The cell survival rate of the PBS-treated group was set at 100%. The cell viability was 25.9 ± 2.2% in the glutamate-treated group and 97.2 ± 1.2% in EGCG-treated group. The cell viability of the glutamate and EGCG co-treated group showed a dose-dependent change in EGCG. It was 40.3 ± 3.9, 61.7 ± 3.3, and 77.4 ± 4.6 at doses of EGCG 10, 20, and 40 μM, respectively (Fig 3B). Glutamate increased intracellular calcium concentration and EGCG co-treatment alleviated this increase (Fig 3C). Intracellular calcium concentration was 481.2 ± 26.4 nM and 101.2 ± 5.3 nM in the glutamate- and EGCG-treated groups, respectively. EGCG co-treatment dose-dependently attenuated the increase caused by glutamate. The calcium concentration in co-treated group was 412.3 ± 35.2 nM, 263.6 ± 21.8 nM, and 147.2 ± 18.9 nM at doses of EGCG 10, 20, and 40 μM, respectively (Fig 3D).

### EGCG alleviates the reduction in hippocalcin expression in glutamate-exposed neuron

**Fig 4:**
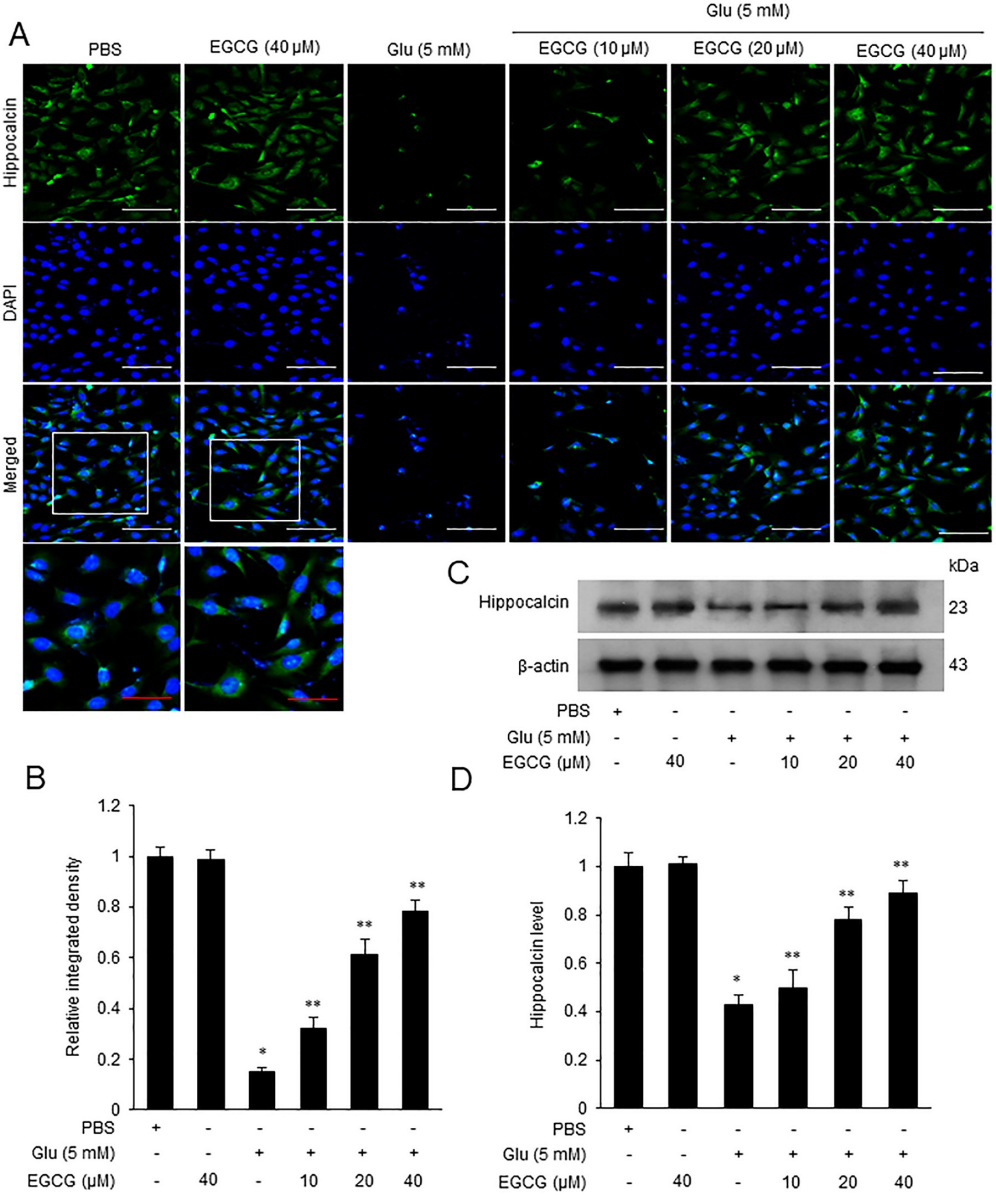
Expression change of hippocalcin in glutamate-exposed neurons. EGCG prevents the reduction in hippocalcin expression in the glutamate-exposed neurons. Representative images of double immunofluorescence labeling (A and B) with hippocalcin (green color) and DAPI (nuclei marker, blue), and Western blot analysis (C and D) and in glutamate (Glu)- and/or epigallocatechin gallate (EGCG)-treated hippocampal neuronal cells (HT22). HT22 cells were treated with glutamate (5 mM) for 24 h and EGCG (10, 20, and 40 μM) was treated 1 h before glutamate exposure. The magnified photos are the enlargement of the square areas. Hippocalcin levels in immunofluorescence labeling were represented as the relative integrated density of each group to that of the integrated density of PBS-treated group. Densitometric analysis is presented as the ratio of hippocalcin intensity to β-actin intensity (D). Scale bars = 50 μm (red), 100 μm (white). The result of PBS-treated group was set to 1. Data (*n* = 5) are presented as means ± S.E.M. * p < 0.001, vs. PBS; ** p < 0.001, vs. Glu (5 mM).

Immunocytochemical staining showed that most cells in the PBS-treated group appeared to be positive cells in response to the hippocalcin (Fig 4A). However, glutamate exposure significantly decreased the number of hippocalcin-positive cells. EGCG co-treatment attenuated this decrease in a dose-dependent manner. Magnified photos showed the localization of hippocalcin in cytoplasm. The relative integrated density in PBS-treated group was set to 1. Hippocalcin levels were 0.15 ± 0.02 in glutamate-treated group. In glutamate and EGCG co-treated group, hippocalcin levels were 0.32 ± 0.04, 0.62 ± 0.05, and 0.78 ± 0.04 at doses of 10, 20, and 40 μM of EGCG, respectively (Fig 4B). The result of Western blot analysis showed the changes of hippocalcin expression in the glutamate- and EGCG-treated groups. Glutamate treatment reduced the expression of hippocalcin and EGCG co-treatment alleviated this decrease in a dose-dependent manner (Fig 4C). The level of hippocalcin protein was 0.43 ± 0.04 in the glutamate-treated group. These levels in the glutamate and EGCG co-treated groups were 0.50 ± 0.05, 0.78 ± 0.06, and 0.89 ± 0.05 at doses of 10, 20, and 40 μM of EGCG, respectively (Fig 4D).

### Hippocalcin siRNA-transfection reduces the expression of hippocalcin

**Fig 5:**
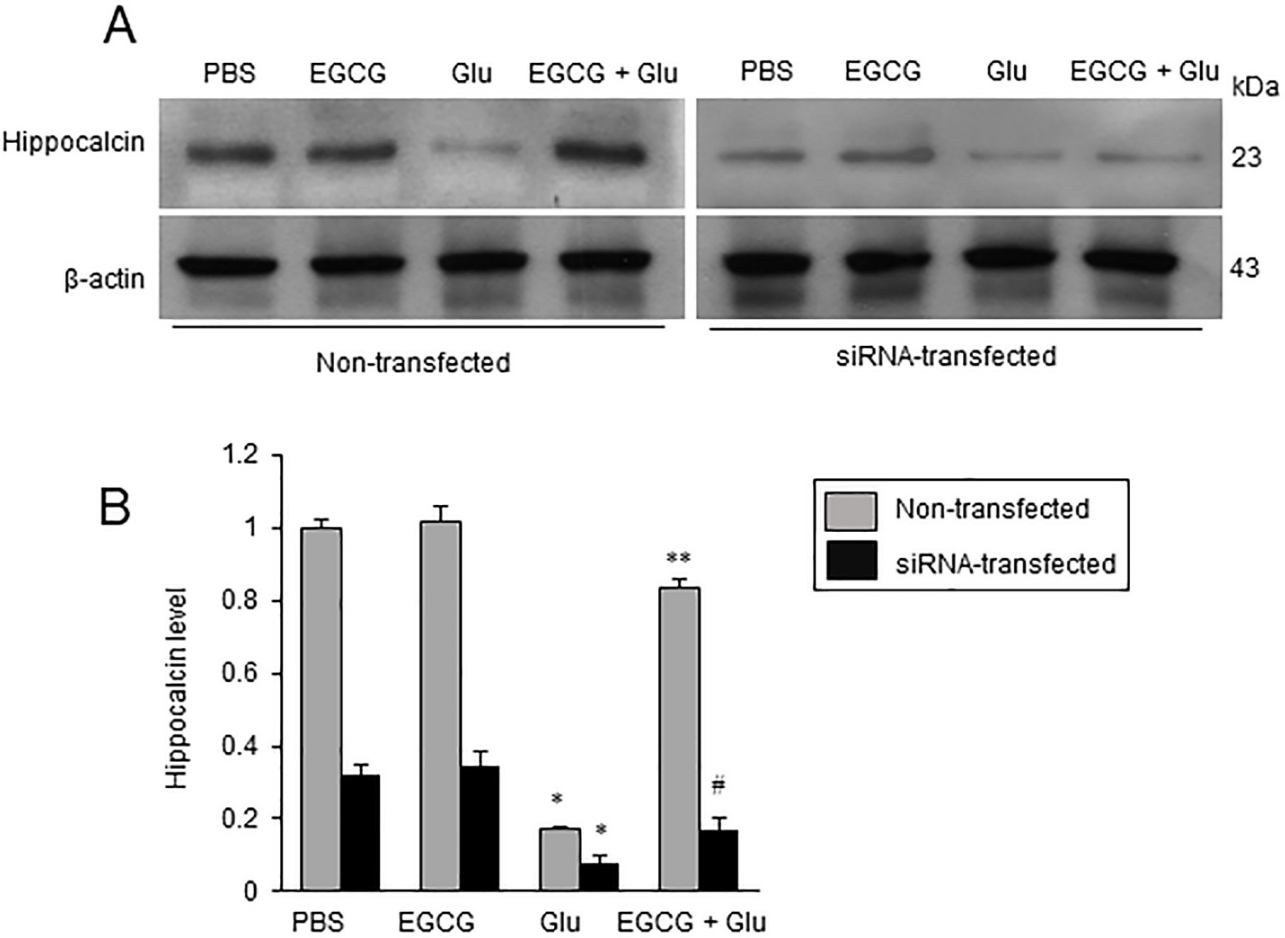
Western blot analysis of hippocalcin in glutamate-exposed neurons. Hippocalcin expression decreases in hippocalcin siRNA-transfected cells. Western blot analysis of hippocalcin (A) in glutamate (Glu)- and/or epigallocatechin gallate (EGCG)-treated hippocampal neuronal cells (HT22) in non- and hippocalcin siRNA-transfected cells. Glutamate (5 mM) was exposed for 24 h and EGCG (40 μM) was treated 1 h before glutamate exposure. Data are presented as the ratio of hippocalcin intensity to β-actin intensity (B). The result of PBS-treated group in non-transfected cells was set to 1. Data (*n* = 5) are presented as means ± S.E.M. * p < 0.01, vs PBS; ** p < 0.001, vs. Glu (5 mM); # p < 0.01, vs. Glu (5 mM).

Hippocalcin siRNA-transfection was performed to investigate the action of hippocalcin in the apoptotic process. Hippocalcin expression in the siRNA-transfected group was decreased compared to the non-transfected group (Fig 5A). EGCG alleviated glutamate-induced a decrease in hippocalcin. The mitigation effect of EGCG was more effective in non-transfected cells than in hippocalcin siRNA-transfected cells. In non-transfected cells, hippocalcin expression level was 0.17 ± 0.02 in the glutamate-treated group and 0.84 ± 0.03 in the EGCG co-treated groups (Fig 5B). Hippocalcin level in siRNA-transfected cells was 0.07 ± 0.02 and 0.16 ± 0.02 in glutamate- and EGCG co-treated groups, respectively (Fig 5B). Thus, we confirmed inhibition of hippocalcin expression by siRNA transfection.

### EGCG attenuates the changes in Bcl-2 and Bax in glutamate-exposed neuron

**Fig 6:**
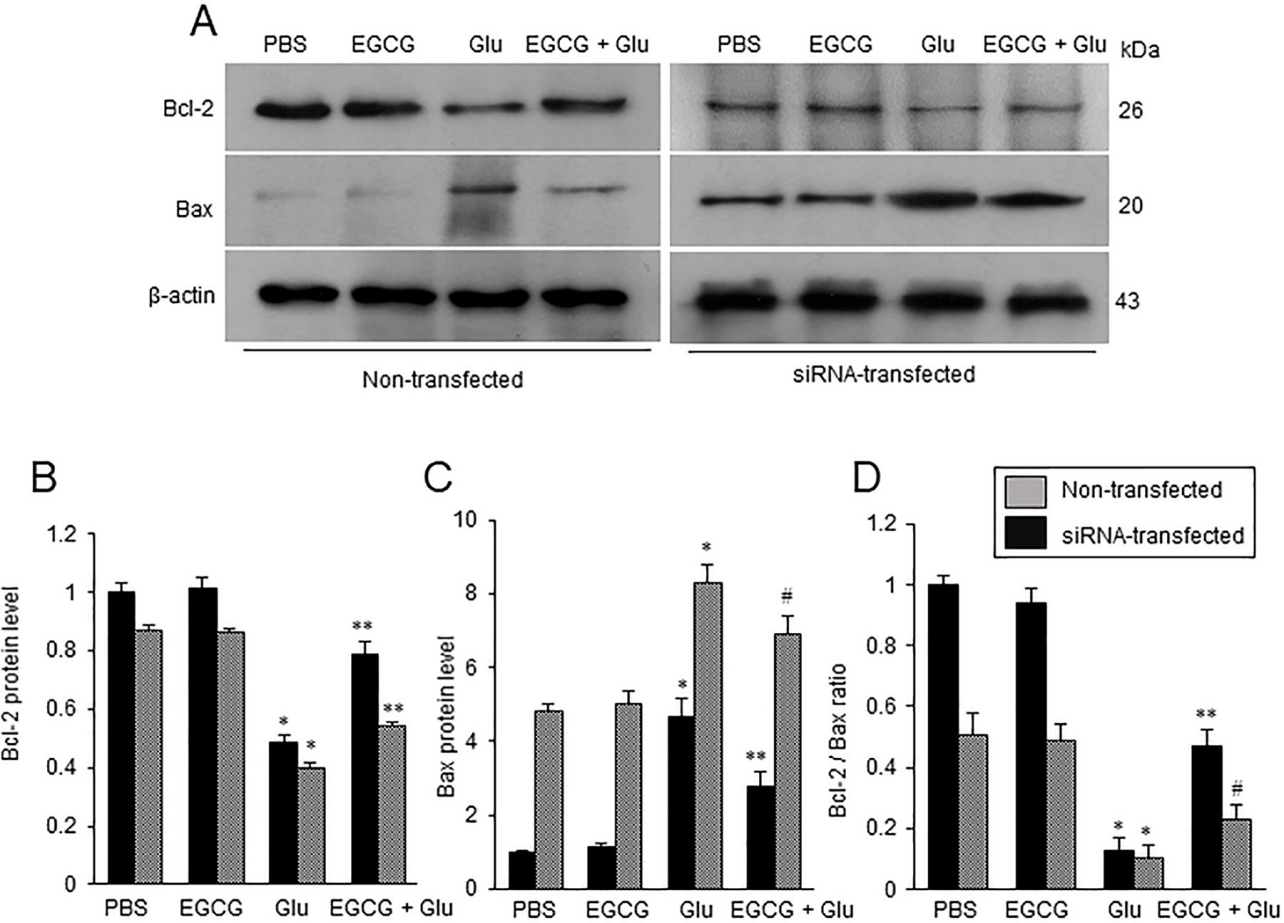
Western blot analysis of Bcl-2 and Bax in hippocalcin siRNA transfected neurons. EGCG attenuates the changes in Bcl-2 and Bax in the glutamate-exposed neurons. Western blot analysis of Bcl-2 and Bax in glutamate (Glu)- and/or epigallocatechin gallate (EGCG)-treated hippocampal neuronal cells (HT22) with or without hippocalcin siRNA transfection (A). Glutamate (5 mM) was exposed for 24 h and EGCG (40 μM) was treated 1 h before glutamate exposure. Densitometric analysis is presented as the ratio of Bcl-2 or Bax intensity to β-actin intensity (B-D). The result of PBS-treated group was set to 1. Data (*n* = 5) are presented as means ± S.E.M. * p < 0.001, vs PBS; ** p < 0.001, vs. Glu (5 mM); # p < 0.01, vs. Glu (5 mM).

The changes in Bcl-2 and Bax expressions were investigated in both non-transfected and siRNA-transfected cells (Fig 6A). Glutamate exposure decreased Bcl-2 expression and increased Bax expression. However, EGCG co-treatment significantly attenuated these changes. These changes were observed in both non-transfected and transfected cells. Bcl-2 expression level in hippocalcin siRNA-transfected cells was lower than in non-transfected cells. Bax expression level in transfected cells was higher than in non-transfected cells. The migration effect of these changes by EGCG was less effective in hippocalcin siRNA-transfected cells than in non-transfected cells. In non-transfected cells, Bcl-2 expression level was 0.49 ± 0.03 in the glutamate-treated group and 0.79 ± 0.04 in the EGCG co-treated groups (Fig 6B). Bcl-2 level in siRNA-transfected cells was 0.41 ± 0.02 and 0.54 ± 0.01 in glutamate- and EGCG co-treated groups, respectively (Fig 6B). In non-transfected cells, Bax expression level was 4.67 ± 0.43 and 2.80 ± 0.39 in glutamate- and EGCG co-treated groups, respectively (Fig 6C). In siRNA-transfected cells, Bax expression level was 8.32 ± 0.73 in the glutamate-treated group and 7.22 ± 0.64 in the glutamate and EGCG co-treated group (Fig 6C). The ratio of Bcl-2 expression and Bax expression was also investigated. It decreased in the glutamate-treated group and this decrease was alleviated in the EGCG co-treated group (Fig 6D). The ratio of these proteins in transfected cells was lower than in non-transfected cells. The ratio of these proteins in non-transfected cells was 0.12 ± 0.04 in the glutamate group and 0.47 ± 0.05 in the EGCG co-treated groups (Fig 6D). The ratio was 0.10 ± 0.04 and 0.23 ± 0.05 in the glutamate- and EGCG co-treated groups with siRNA transfection (Fig 6D).

### EGCG prevents changes of caspase-3 and cleaved caspase-3 in glutamate exposed neuron

**Fig 7:**
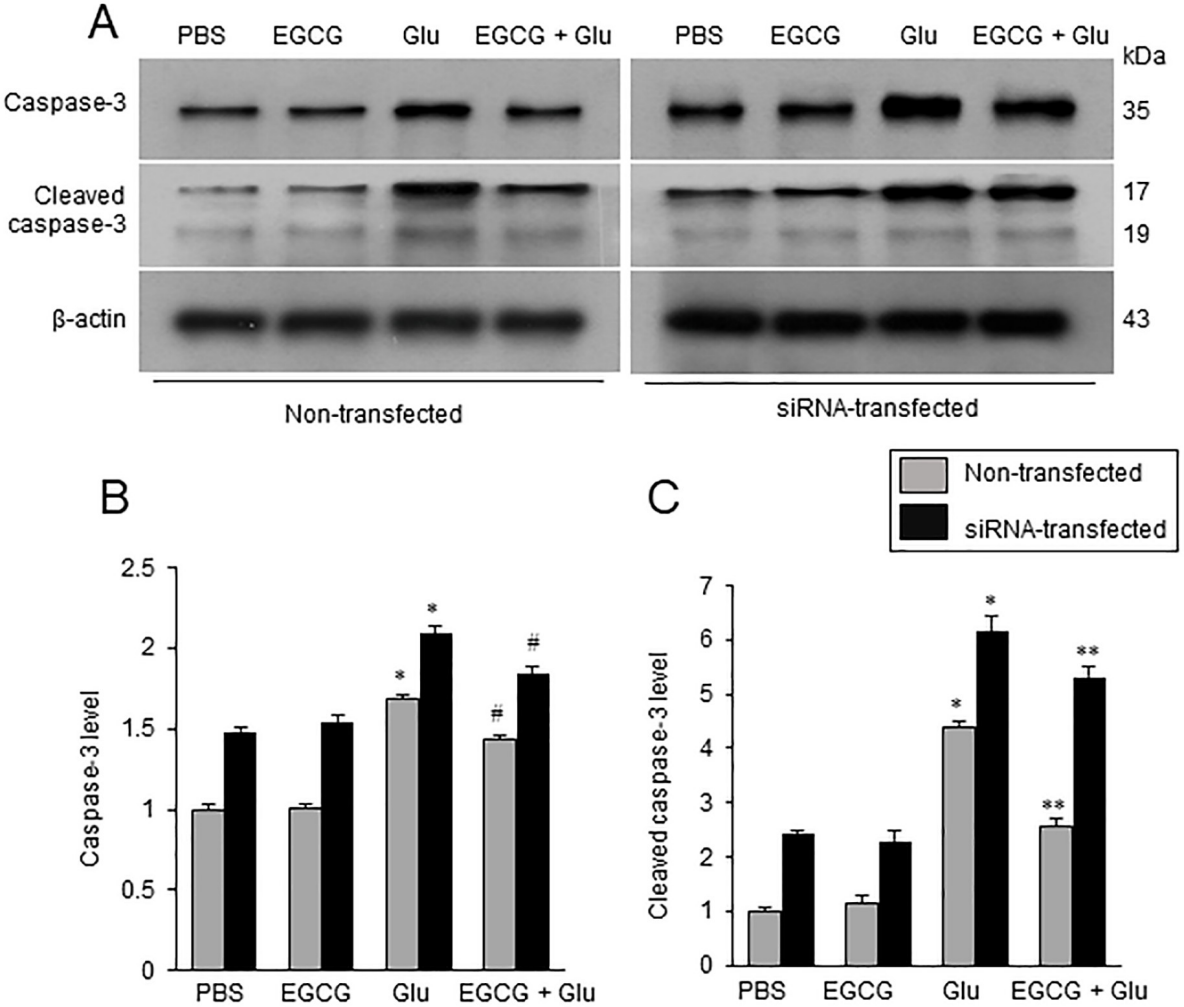
Western blot analysis of caspase-3 and cleaved caspase-3 in hippocalcin siRNA transfected neurons. EGCG attenuates the changes in caspase-3 and cleaved caspase-3 in the glutamate-exposed neuron. Western blot analysis of caspase-3 and cleaved caspase-3 in glutamate (Glu)- and/or epigallocatechin gallate (EGCG)-treated hippocampal neuronal cells (HT22) in non- and hippocalcin siRNA-transfected cells (A). Glutamate (5 mM) was exposed for 24 h and EGCG (40 μM) was treated 1 h before glutamate exposure. Densitometric analysis is presented as the ratio of caspase-3 or cleaved caspase-3 intensity to β-actin intensity (B, C). The result of PBS-treated group was set to 1. Data (*n* = 5) are presented as means ± S.E.M. * p < 0.001, vs. PBS; ** p < 0.001, vs. Glu (5 mM); ** p < 0.01, vs. Glu (5 mM).

Glutamate treatment increased caspase-3 and cleaved caspase-3 expression. EGCG co-treatment alleviated these increases in both non-transfected and transfected groups (Fig 7A). siRNA-transfected cells showed greater caspase-3 and cleaved-caspase-3 expression than non-transfected cells. Caspase-3 expression level in non-transfected cells was 1.69 ± 0.02 in the glutamate- group and 1.43 ± 0.02 in the glutamate and EGCG co-treated group (Fig 7B). In hippocalcin siRNA-transfected cell, caspase-3 expression level was 2.09 ± 0.04 and 1.85 ± 0.03 in the glutamate- and EGCG co-treated groups, respectively (Fig 7B). Cleaved caspase-3 expression level in non-transfected cells was 4.39 ± 0.10 in glutamate- and 2.57 ± 0.13 in EGCG co-treated group (Fig 7C). Cleaved caspase-3 expression level in the transfected cells was 6.15 ± 0.35 in glutamate- and 5.32 ± 0.21 in EGCG co-treated group (Fig 7C).

## Discussion

This study confirmed that EGCG improves neuronal dysfunctions in ischemic stroke. EGCG attenuates the impairment of movement and the disorders of memory and learning in ischemic stroke [27, 35]. We previously demonstrated that EGCG reduces ischemic lesions through the regulation of apoptotic signaling pathways [32]. Cerebral ischemia leads to neurobehavioral deficits in sensorimotor function and abnormal clinical signs such as seizures and unconsciousness. It induces severe infarction and histopathological changes. EGCG improves neurological behavior disorders caused by MCAO damage and attenuates histopathological changes. We treated 50 mg/kg of EGCG in rats according to previous reports [26, 27]. This dosage in rats is equivalent to nearly 4 mg/kg in humans [36]. A previous study has shown that clinical trials have demonstrated the safety of 10 mg/kg EGCG [37]. Therefore, it is considered that the dose of EGCG administered is safe and has a protective effect against brain damage. Moreover, various substances provide neuroprotective effects against ischemic injury by reducing oxidative stress and inhibiting neuroinflammation [38, 39]. We revealed that EGCG exerts neuroprotective effects by modulating various proteins [40]. This study focused on the expression of hippocalcin protein by EGCG in animal models of ischemic stroke. We confirmed a decrease in hippocalcin in MCAO animals using a variety of techniques including proteomics studies, reverse transcription-PCR, and Western blot analysis. This study clearly showed that EGCG attenuates MCAO damage-induced reduction of hippocalcin. Hippocalcin exerts an anti-apopototic effect and performs neuroprotective function against neuronal damage [17, 19]. Thus, we demonstrate that EGCG exerts a neuroprotective effect by regulating hippocalcin in cerebral ischemia.

Oxidative stress is a major mechanism that governs neuronal cell death in ischemic injury [41]. It causes disruption of calcium channels distributed in the cell membrane and endoplasmic reticulum, leading to changes in intracellular calcium levels within neurons [42]. The accumulation of calcium ions within the mitochondria leads to permeable transposable pore formation and ultimately to the release of cytochrome c, which causes mitochondrial dysfunction and activates caspase cascade [43]. In addition, calcium acts as a direct activator of calpain protease, a calcium-dependent enzyme. Activation of this enzyme initiates a process of degradation of structural and enzymatic proteins that play an important role in cellular homeostasis and survival [44]. The degradation of these important cellular components adversely affects cell survival and eventually leads to apoptosis.

Glutamate-mediated neurotoxicity is one of the major pathophysiological mechanisms of ischemic brain injury [45]. It increases intracellular calcium levels in neurons, and reduces plasma membrane calcium pump activity, which can cause neuronal cell death [46]. The increase in calcium level initiates apoptosis-related signaling pathways and activates degradative enzymes, leading to neuronal death [44]. We have previously demonstrated that excessive exposure to glutamate leads to neuronal cell death [47]. This study confirms that EGCG dose-dependently attenuates neuronal cell death and intracellular calcium overload in glutamate-exposed neurons. Hippocalcin contributes to neuronal calcium homeostasis and protects hippocampal neurons against excitatory toxins by enhancing calcium extrusion [48]. Hippocalcin performs a neuroprotective function by reducing intracellular calcium concentration in neurological damage. Hippocalcin has neuroprotective effect on excitatory toxicity by interacting with NAIP [14, 15]. Moreover, calcium-binding proteins regulate the Ras/Raf/MEK/ERK pathway, which regulates cell cycle progression and cell survival [49]. Hippocalcin acts as an important factor in the activation of Raf, which is conducted by Ras [50]. These results suggest that the Ras/Raf/MEK/ERK pathway is involved in the inhibition of apoptosis of hippocalcin. Our results demonstrated that EGCG has neuroprotective effects against glutamate excitatory toxicity through regulation of hippocalcin expression and intracellular calcium concentration, which is involved in apoptosis and cell survival signaling.

**Fig 8:**
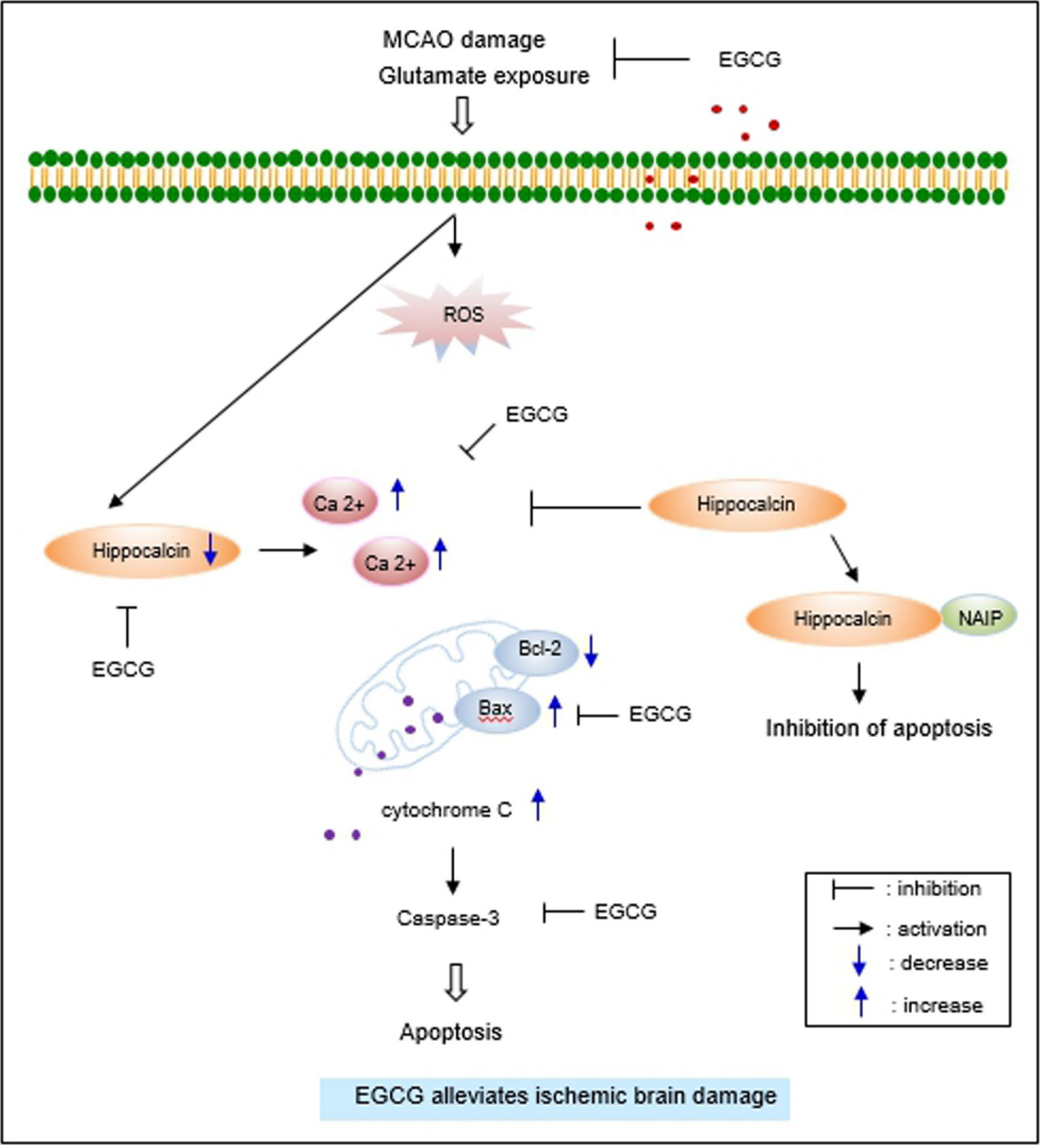
The neuroprotective mechanism of EGCG on MCAO damage and glutamate toxicity.

We used a siRNA-transfection technique to investigate whether EGCG exerts neuroprotective effects through the regulation of hippocalcin and apoptosis-related proteins in glutamate-exposed cells. We showed that hippocalcin-silenced cells were more vulnerable to glutamate-induced apoptosis than non-transfected cells. Moreover, hippocalcin knockout mice are more susceptible to neuronal damage than hippocalcin +/+ mice [49]. Neuroprotective effect of EGCG on glutamate exposure was weakened in the hippocalcin-suppressed condition. These studies demonstrated that hippocalcin contributes to neuroprotection against neuronal damage. We also investigated the changes in Bcl-2 family proteins and caspase-3 expressions in a hippocalcin-suppressed condition. The decrease of Bcl-2 and the increase of Bax are important events in the induction of apoptosis. The reduction of the Bcl-2/Bax ratio is considered an essential process in inducing apoptosis. Glutamate exposure decreased the anti-apoptotic protein Bcl-2 and increased pro-apoptotic Bax. It also induced a decrease in the Bcl-2/Bax ratio. EGCG treatment in glutamate-exposed cells alleviated a decrease in the Bcl-2/Bax ratio. The mitigation effect of EGCG was more pronounced in non-transfected cells than in hippocalcin siRNA transfected cells. Hippocalcin acts as an important protein for the regulation of Bcl-2 family proteins and modulates anti-apoptotic functions during neuronal cell damage. Thus, these results can demonstrate that EGCG regulates the expression of Bcl-2 family proteins in glutamate-induced apoptosis by controlling hippocalcin. Furthermore, the expressions of caspase-3 and cleaved caspase-3 were increased in glutamate-exposed conditions, which was attenuated by EGCG treatment. The alleviative effect of EGCG was greater in non-transfected cells than in hippocalcin siRNA-transfected cells. Hippocalcin exerts neuroprotective effects against calcium-induced cell death through the activation of caspase-3 [14]. These results showed that hippocalcin contributes to the anti-apoptotic process through the regulation of Bcl-2 protein and caspase-3. Fig 8 schematized the neuroprotective mechanisms of EGCG against MCAO damage and glutamate toxicity.

## Conclusion

The results of this study revealed the relation between EGCG and hippocalcin and the regulation of intracellular calcium by EGCG in ischemic brain injury. Our findings showed that EGCG protects neurons against neuronal damage through the regulation of hippocalcin and apoptosis-related proteins. We provided evidence that EGCG has a neuroprotective effect by controlling the expression of hippocalcin and modulating apoptosis in ischemic neuronal damage. Furthermore, we suggest that EGCG has potential as a promising candidate for the treatment of ischemic stroke.

## Supporting information

S1 File (ZIP)

S1 Data (XLSX)
