# Supplementary figures and images for: Epigallocatechin gallate improves neuronal damage in animal model of ischemic stroke and glutamate-exposed neurons via modulation of hippocalcin expression
Source: PLoS One. 2024 Mar 1;19(3):e0299042. doi: 10.1371/journal.pone.0299042 (PMC10906901; doi:10.1371/journal.pone.0299042)

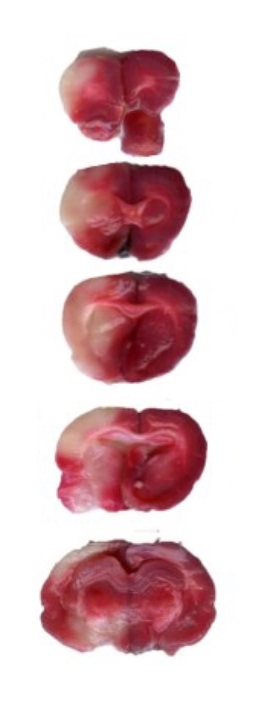

Supplement: S1 File — (ZIP) [file pone.0299042.s001.zip › Figure 1C EGCG+MCAO.jpg]

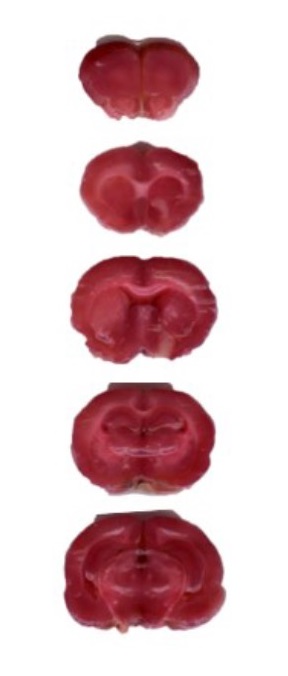

Supplement: S1 File — (ZIP) [file pone.0299042.s001.zip › Figure 1C EGCG+Sham.jpg]

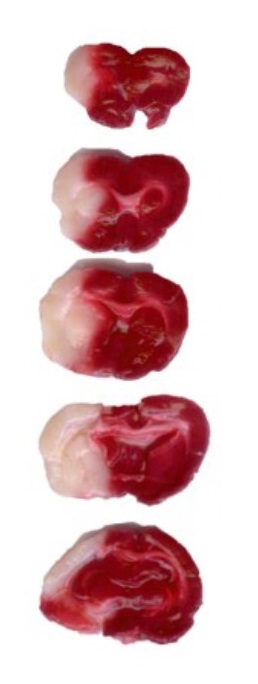

Supplement: S1 File — (ZIP) [file pone.0299042.s001.zip › Figure 1C PBS+MCAO.jpg]

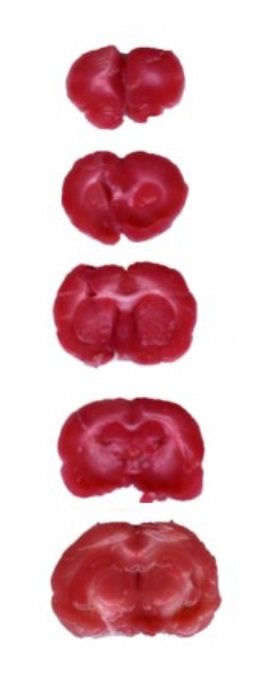

Supplement: S1 File — (ZIP) [file pone.0299042.s001.zip › Figure 1C PBS+Sham.jpg]

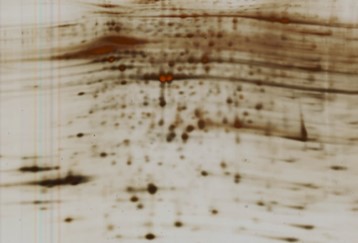

Supplement: S1 File — (ZIP) [file pone.0299042.s001.zip › Figure 2A EGCG+MCAO.jpg]

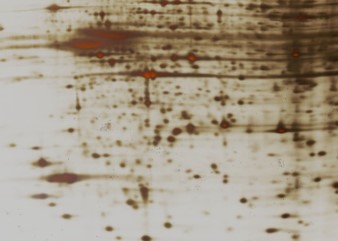

Supplement: S1 File — (ZIP) [file pone.0299042.s001.zip › Figure 2A EGCG+Sham.jpg]

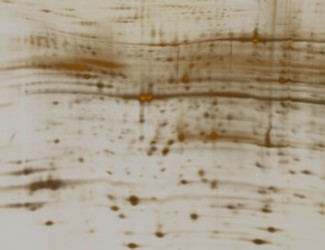

Supplement: S1 File — (ZIP) [file pone.0299042.s001.zip › Figure 2A PBS+MCAO.jpg]

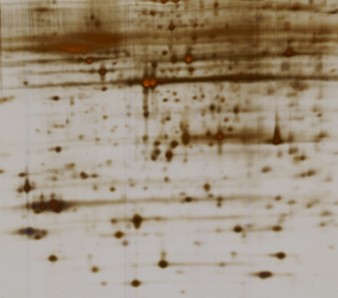

Supplement: S1 File — (ZIP) [file pone.0299042.s001.zip › Figure 2A PBS+Sham.jpg]

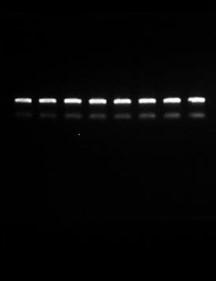

Supplement: S1 File — (ZIP) [file pone.0299042.s001.zip › Figure 2C beta-actin PCR.jpg]

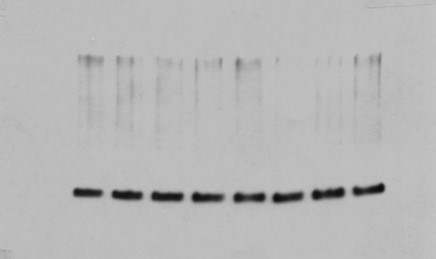

Supplement: S1 File — (ZIP) [file pone.0299042.s001.zip › Figure 2C beta-actin Western blot.jpg]

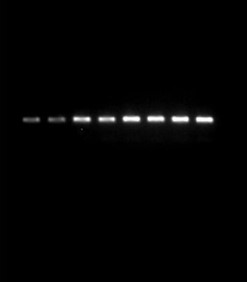

Supplement: S1 File — (ZIP) [file pone.0299042.s001.zip › Figure 2C Hippocalcin PCR.jpg]

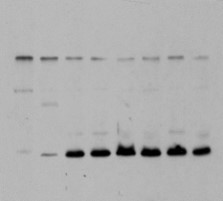

Supplement: S1 File — (ZIP) [file pone.0299042.s001.zip › Figure 2C Hippocalcin Western blot.jpg]

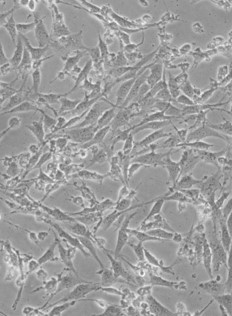

Supplement: S1 File — (ZIP) [file pone.0299042.s001.zip › Figure 3A EGCG(10uM)+Glu.jpg]

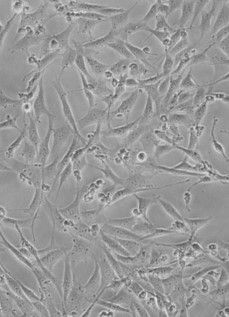

Supplement: S1 File — (ZIP) [file pone.0299042.s001.zip › Figure 3A EGCG(20uM)+Glu.jpg]

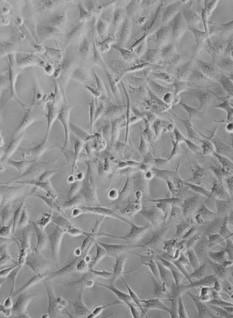

Supplement: S1 File — (ZIP) [file pone.0299042.s001.zip › Figure 3A EGCG(40uM).jpg]

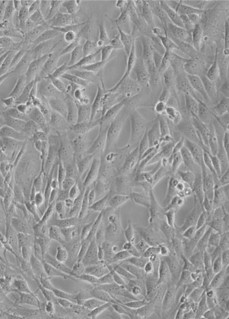

Supplement: S1 File — (ZIP) [file pone.0299042.s001.zip › Figure 3A EGCG(40uM)+Glu.jpg]

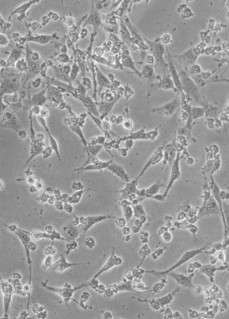

Supplement: S1 File — (ZIP) [file pone.0299042.s001.zip › Figure 3A Glu.jpg]

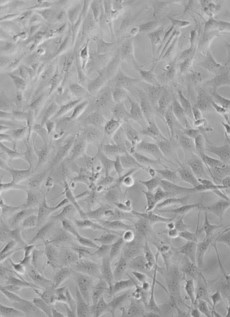

Supplement: S1 File — (ZIP) [file pone.0299042.s001.zip › Figure 3A PBS.jpg]

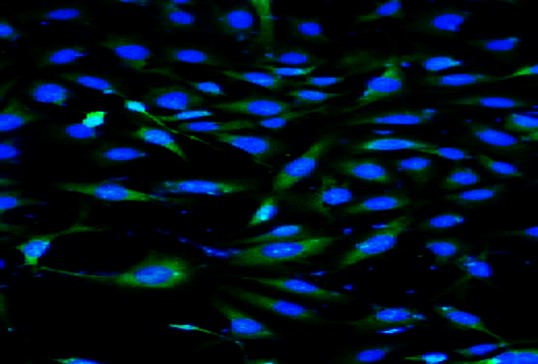

Supplement: S1 File — (ZIP) [file pone.0299042.s001.zip › Figure 4A EGCG(40uM) Merged.jpg]

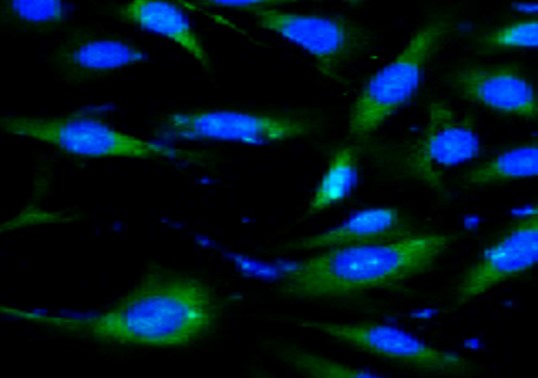

Supplement: S1 File — (ZIP) [file pone.0299042.s001.zip › Figure 4A EGCG(40uM).jpg]

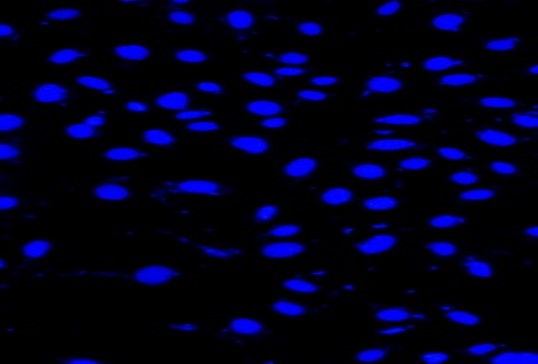

Supplement: S1 File — (ZIP) [file pone.0299042.s001.zip › Figure 4A EGCG(40uM)+DAPI.jpg]

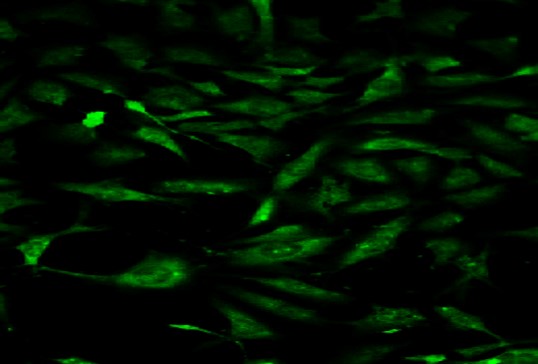

Supplement: S1 File — (ZIP) [file pone.0299042.s001.zip › Figure 4A EGCG(40uM)+Hippocalcin.jpg]

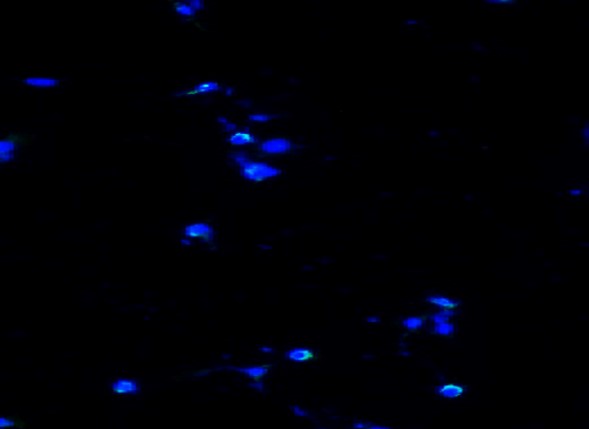

Supplement: S1 File — (ZIP) [file pone.0299042.s001.zip › Figure 4A Glu(5mM) Merged.jpg]

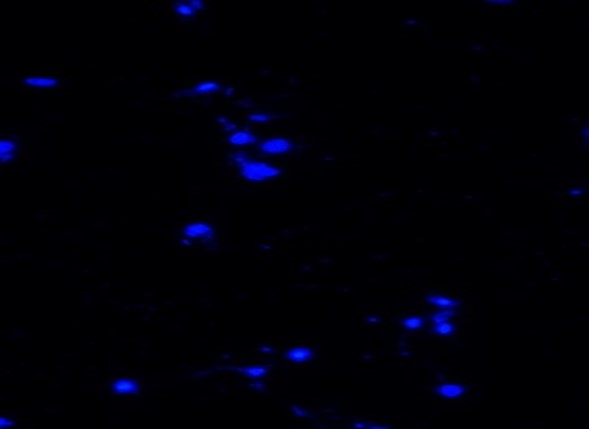

Supplement: S1 File — (ZIP) [file pone.0299042.s001.zip › Figure 4A Glu(5mM)+DAPI.jpg]

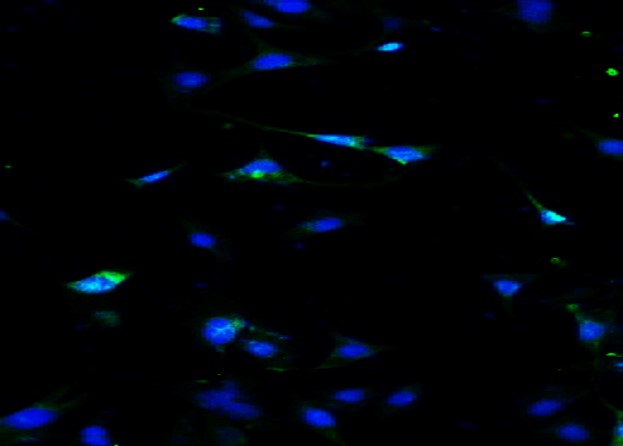

Supplement: S1 File — (ZIP) [file pone.0299042.s001.zip › Figure 4A Glu(5mM)+EGCG(10uM) Merged.jpg]

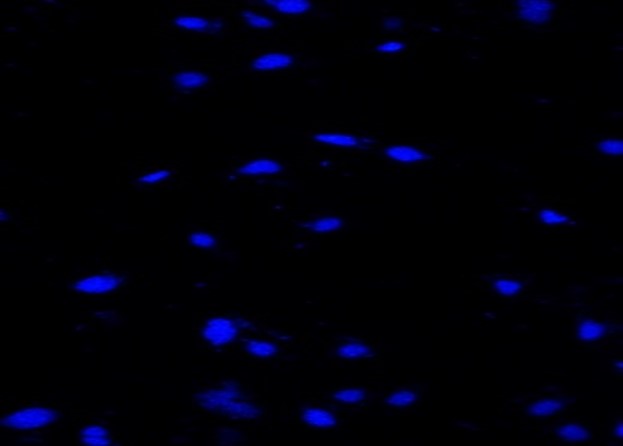

Supplement: S1 File — (ZIP) [file pone.0299042.s001.zip › Figure 4A Glu(5mM)+EGCG(10uM)+DAPI.jpg]

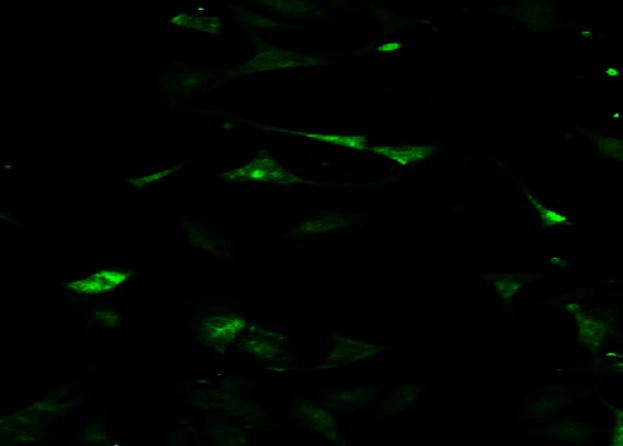

Supplement: S1 File — (ZIP) [file pone.0299042.s001.zip › Figure 4A Glu(5mM)+EGCG(10uM)+Hippocalcin.jpg]

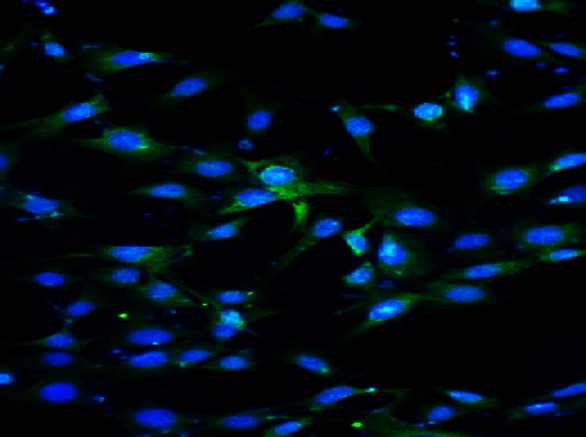

Supplement: S1 File — (ZIP) [file pone.0299042.s001.zip › Figure 4A Glu(5mM)+EGCG(20uM) Merged.jpg]

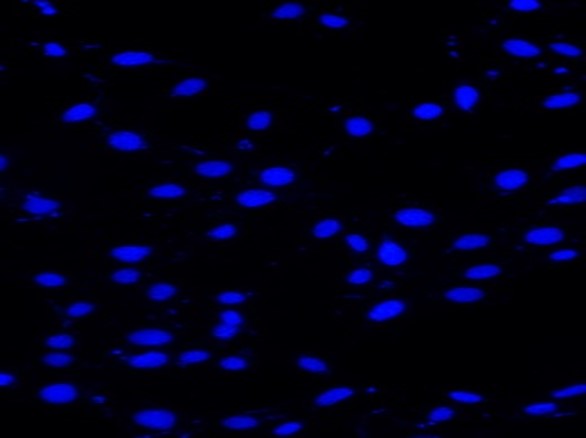

Supplement: S1 File — (ZIP) [file pone.0299042.s001.zip › Figure 4A Glu(5mM)+EGCG(20uM)+DAPI.jpg]

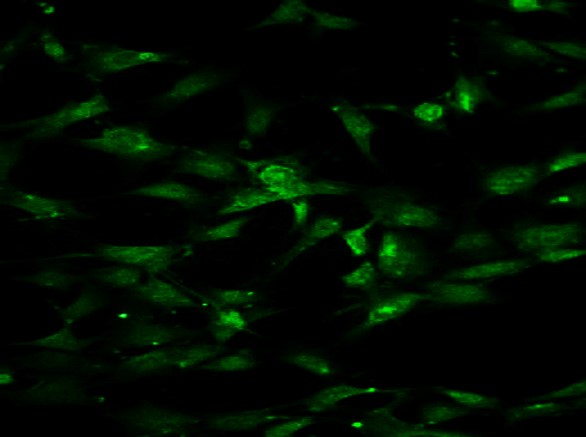

Supplement: S1 File — (ZIP) [file pone.0299042.s001.zip › Figure 4A Glu(5mM)+EGCG(20uM)+Hippocalcin.jpg]

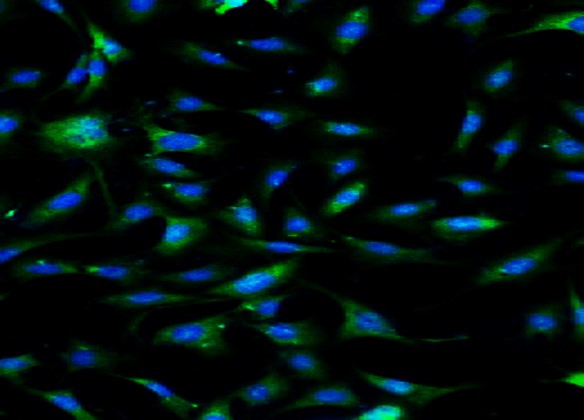

Supplement: S1 File — (ZIP) [file pone.0299042.s001.zip › Figure 4A Glu(5mM)+EGCG(40uM) Merged.jpg]

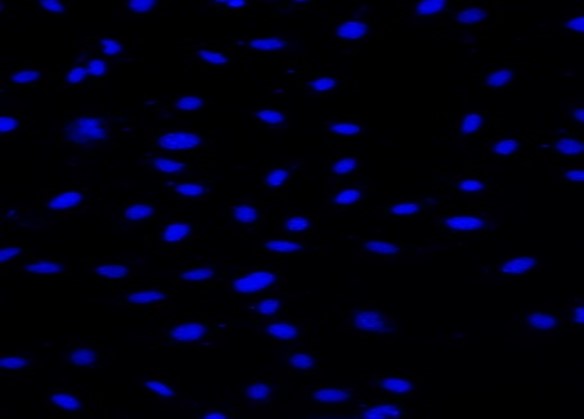

Supplement: S1 File — (ZIP) [file pone.0299042.s001.zip › Figure 4A Glu(5mM)+EGCG(40uM)+DAPI.jpg]

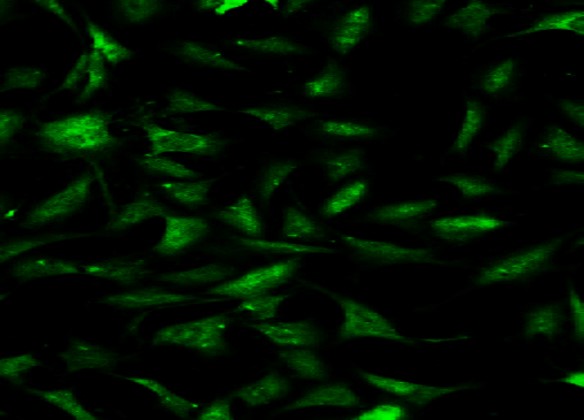

Supplement: S1 File — (ZIP) [file pone.0299042.s001.zip › Figure 4A Glu(5mM)+EGCG(40uM)+Hippocalcin.jpg]

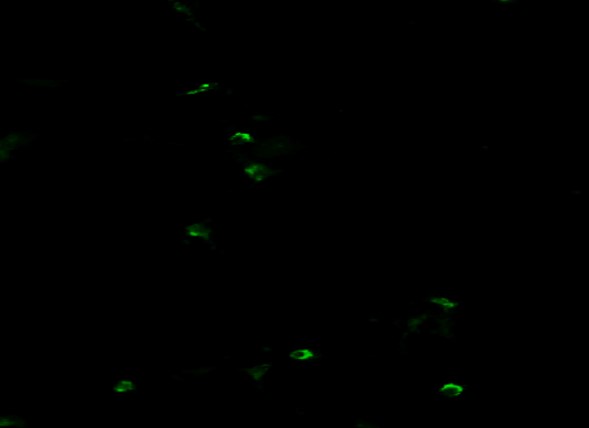

Supplement: S1 File — (ZIP) [file pone.0299042.s001.zip › Figure 4A Glu(5mM)+Hippocalcin.jpg]

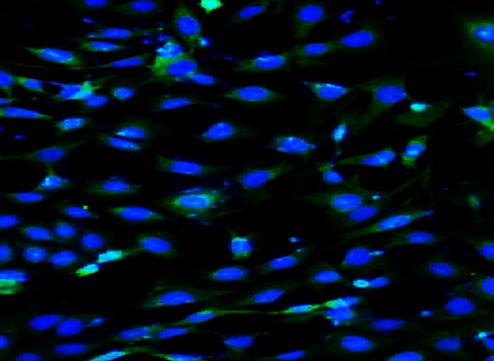

Supplement: S1 File — (ZIP) [file pone.0299042.s001.zip › Figure 4A PBS Merged.jpg]

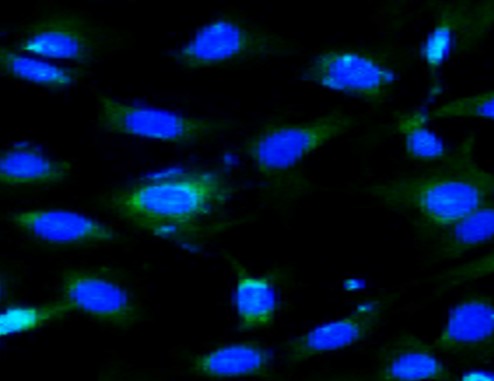

Supplement: S1 File — (ZIP) [file pone.0299042.s001.zip › Figure 4A PBS.jpg]

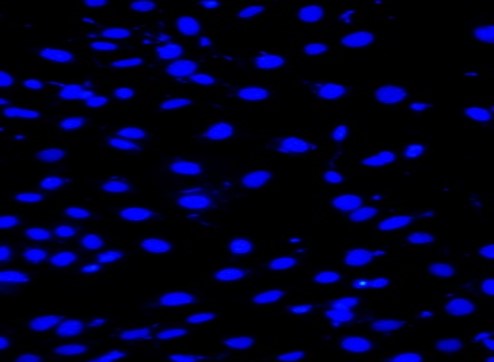

Supplement: S1 File — (ZIP) [file pone.0299042.s001.zip › Figure 4A PBS+DAPI.jpg]

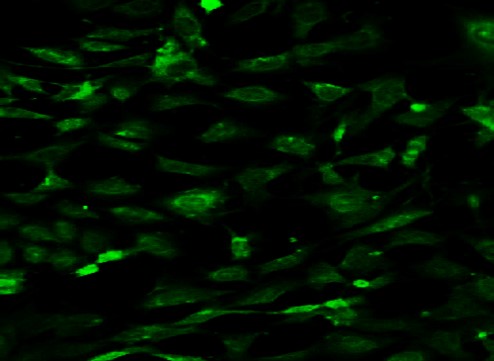

Supplement: S1 File — (ZIP) [file pone.0299042.s001.zip › Figure 4A PBS+Hippocalcin.jpg]

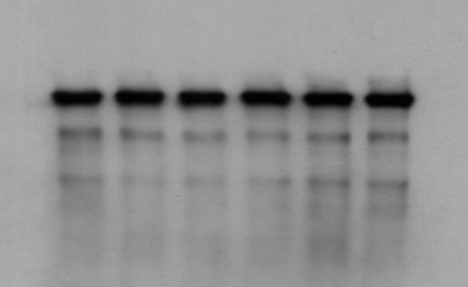

Supplement: S1 File — (ZIP) [file pone.0299042.s001.zip › Figure 4C beta-actin.jpg]

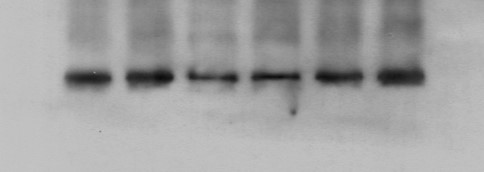

Supplement: S1 File — (ZIP) [file pone.0299042.s001.zip › Figure 4C Hippocalcin.jpg]

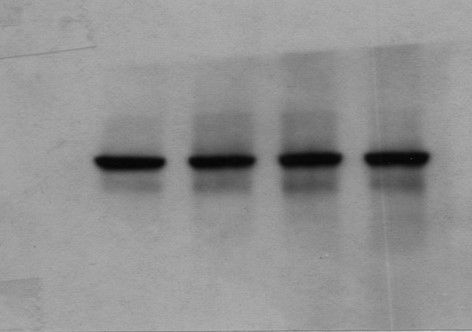

Supplement: S1 File — (ZIP) [file pone.0299042.s001.zip › Figure 5A beta-actin Non-trasnfected.jpg]

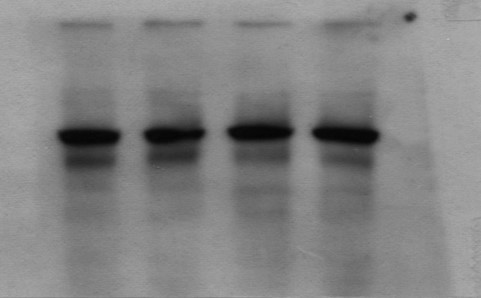

Supplement: S1 File — (ZIP) [file pone.0299042.s001.zip › Figure 5A beta-actin siRNA-transfected.jpg]

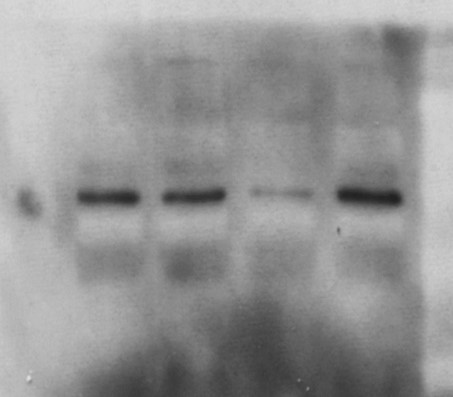

Supplement: S1 File — (ZIP) [file pone.0299042.s001.zip › Figure 5A Hippocalcin Non-trasnfected.jpg]

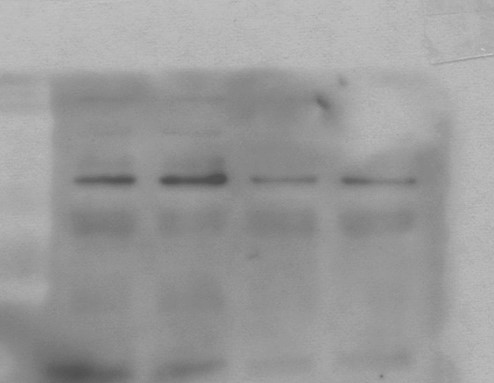

Supplement: S1 File — (ZIP) [file pone.0299042.s001.zip › Figure 5A Hippocalcin siRNA-transfected.jpg]

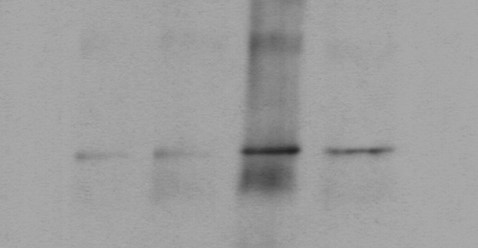

Supplement: S1 File — (ZIP) [file pone.0299042.s001.zip › Figure 6A Bax Non-transfected.jpg]

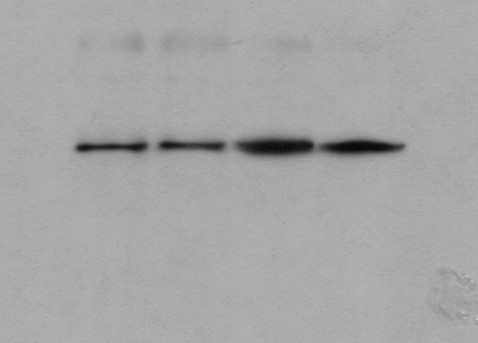

Supplement: S1 File — (ZIP) [file pone.0299042.s001.zip › Figure 6A Bax siRNA-transfected.jpg]

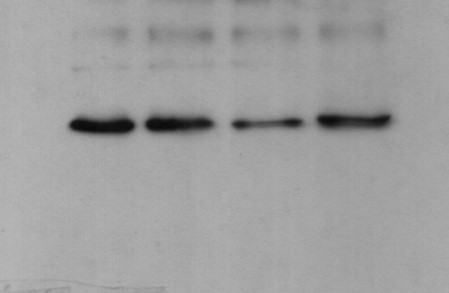

Supplement: S1 File — (ZIP) [file pone.0299042.s001.zip › Figure 6A BCl-2 Non-transfected.jpg]

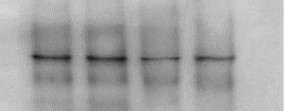

Supplement: S1 File — (ZIP) [file pone.0299042.s001.zip › Figure 6A BCl-2 siRNA-transfected.jpg]

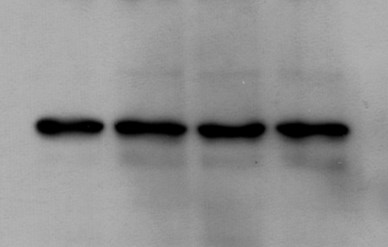

Supplement: S1 File — (ZIP) [file pone.0299042.s001.zip › Figure 6A beta-actin Non-transfected.jpg]

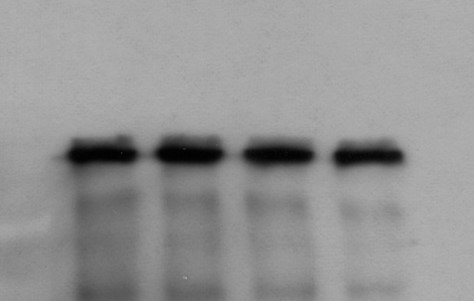

Supplement: S1 File — (ZIP) [file pone.0299042.s001.zip › Figure 6A beta-actin siRNA-transfected.jpg]

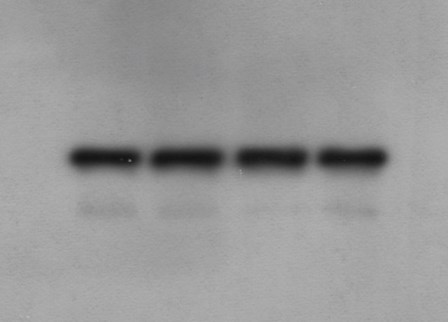

Supplement: S1 File — (ZIP) [file pone.0299042.s001.zip › Figure 7A beta-actin Non-transfected.jpg]

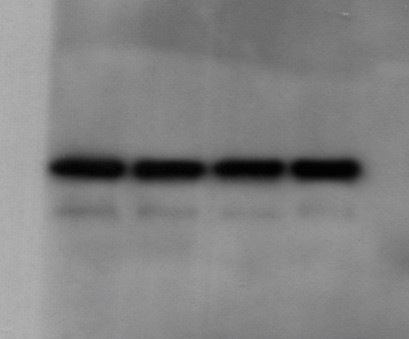

Supplement: S1 File — (ZIP) [file pone.0299042.s001.zip › Figure 7A beta-actin siRNA-transfected.jpg]

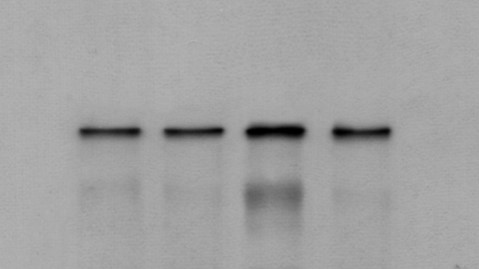

Supplement: S1 File — (ZIP) [file pone.0299042.s001.zip › Figure 7A Caspase-3 Non-transfected.jpg]

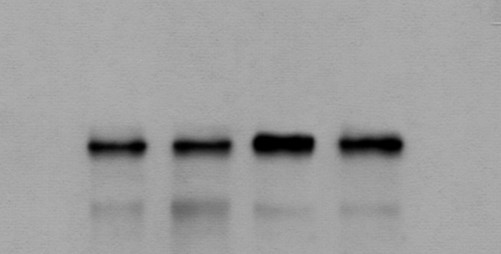

Supplement: S1 File — (ZIP) [file pone.0299042.s001.zip › Figure 7A Caspase-3 siRNA-transfected.jpg]

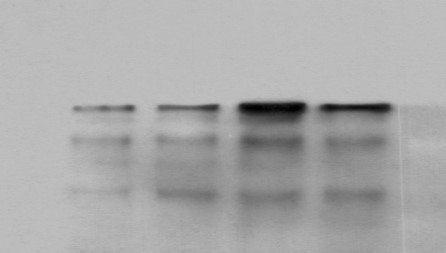

Supplement: S1 File — (ZIP) [file pone.0299042.s001.zip › Figure 7A Cleaved caspase-3 Non-transfected.jpg]

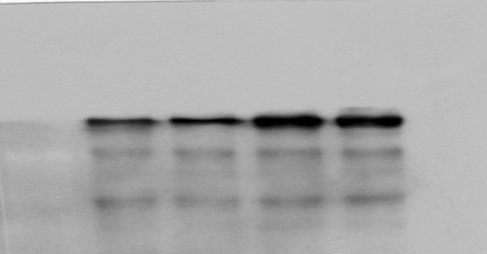

Supplement: S1 File — (ZIP) [file pone.0299042.s001.zip › Figure 7A Cleaved caspase-3 siRNA-transfected.jpg]
